# Normal human craniofacial growth and development from 0 to 4 years

**DOI:** 10.1038/s41598-023-36646-8

**Published:** 2023-06-14

**Authors:** Ce Liang, Antonio Profico, Costantino Buzi, Roman H. Khonsari, David Johnson, Paul O’Higgins, Mehran Moazen

**Affiliations:** 1 Department of Mechanical Engineering, University College London, London, UK; 2 Department of Biology, University of Pisa, Pisa, Italy; 3 Institut Català de Paleoecologia Humana i Evolució Social (IPHES-CERCA), Tarragona, Spain; 4 Departament d’Història i Història de l’Art, Universitat Rovira i Virgili, Tarragona, Spain; 5 Department of Maxillofacial Surgery and Plastic Surgery, Necker – Enfants Malades Hospital, Assistance Publique – Hôpitaux de Paris, Paris, France; 6 Oxford Craniofacial Unit, Oxford University Hospital, Oxford, UK; 7 PalaeoHub, Department of Archaeology, University of York, York, UK; 8 Hull York Medical School, University of York, York, UK

**Keywords:** Developmental biology, Structural biology, Anatomy, Engineering

## Abstract

Knowledge of human craniofacial growth (increase in size) and development (change in shape) is important in the clinical treatment of a range of conditions that affects it. This study uses an extensive collection of clinical CT scans to investigate craniofacial growth and development over the first 48 months of life, detail how the cranium changes in form (size and shape) in each sex and how these changes are associated with the growth and development of various soft tissues such as the brain, eyes and tongue and the expansion of the nasal cavity. This is achieved through multivariate analyses of cranial form based on 3D landmarks and semi-landmarks and by analyses of linear dimensions, and cranial volumes. The results highlight accelerations and decelerations in cranial form changes throughout early childhood. They show that from 0 to 12 months, the cranium undergoes greater changes in form than from 12 to 48 months. However, in terms of the development of overall cranial shape, there is no significant sexual dimorphism in the age range considered in this study. In consequence a single model of human craniofacial growth and development is presented for future studies to examine the physio-mechanical interactions of the craniofacial growth.

## Introduction

Characterising the growth (increase in size) and development (change in shape) of the human craniofacial system has long been of interest from the perspective of understanding how the cranium and its associated soft tissues grow, develop and interact during postnatal ontogeny^1–12^. Knowledge of normal skull growth and development in the first few years of life is of particular relevance to clinical management of various congenital diseases affecting the craniofacial system (e.g. craniosynostosis, caused by early fusion of cranial sutures^13,14^). Treatment of these conditions aims to modify the cranium and its subsequent growth and development to achieve an adult cranial form (size and shape) that is within the normal range of variation^14^. This requires detailed knowledge of normal craniofacial growth, and treatment planning requires understanding of growth interactions within the cranium. In early childhood (0–48 months from birth), the craniofacial system undergoes rapid changes in form, in response to, and to accommodate, the growth and development of various soft tissues such as brain, eyes and tongue and the expansion of the nasal cavity^15^.

Many previous studies have characterised the early growth and development of the facial^15–20^ or neurocranial regions^21–23^. These studies have mainly focused on quantifying the morphological changes of the skull and estimating an average model of the skull at different ages or sizes. Yet to the best of our knowledge few studies have carried out detailed characterisation of changes in volumes of different craniofacial regions (i.e. nasal cavity, upper intraoral volume, orbital volume) during growth. Several of these studies investigated the interplay and effects on craniofacial growth and development of physical factors such as volumetric changes of soft tissues (e.g. the brain or intracranial volume) and the action of muscles, based on relatively small sample sizes^3,15,23–26^. In advancing our fundamental understanding of the craniofacial system, a robust model of skull growth and development and of growth interactions is important because of the potential wider applications. For example, such a model could provide insights into the evolution of the modern human skull^18,27^, or it can advance assessment and treatment planning in conditions such as craniosynostosis where optimum management is still debated^14,28–30^.

**Figure 1:**
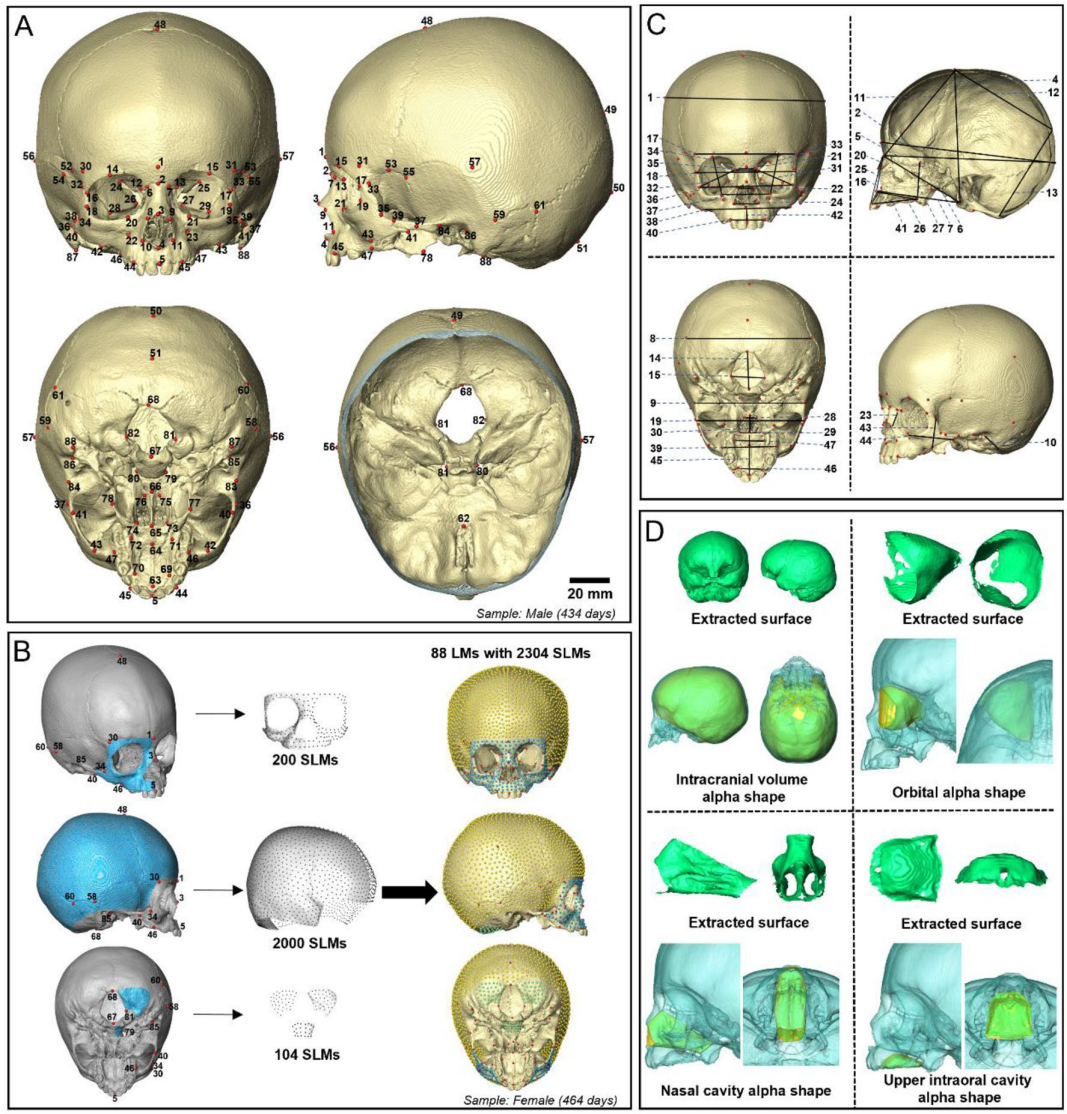
Landmark configurations and craniofacial measurements: (**A**) The template of 88 anatomical landmarks (LMs; in red, see Supplementary Table S1 for detailed definitions). (**B**) Surface semi-landmark (SLM) configuration on the reference skull surface (female, 464 days old). Template surface SLM sets were created by distributing points approximately evenly over segmented surface patches (in blue) defined by the selected LMs (in red; see Supplementary Fig. S1A–C for boundary details). The full configuration consists of 88 LMs (in red) and 2304 SLMs (facial SLMs in blue; neurocranial SLMs in yellow and green). (**C**) 47 linear cranial dimensions (in black) computed between selected LMs (cranial circumference (3), is not shown, it was measured in the plane which passes through the landmark point 1 and 50 and is perpendicular to the midsagittal plane; see Supplementary Table S2A for details of all linear dimensions). (**D**) Four cranial volumes including intracranial volume (ICV), left orbital volume (LOV), nasal cavity volume (NCV) and upper intraoral volume (UIV)—see text and Supplementary Fig. S1D–G for definitions of volumes.

The overall aim of this study is to carry out a detailed characterisation of postnatal human craniofacial ontogeny from birth to 48 months. Specifically, the aims are: (1) to detail how the cranium changes in form (size and shape) from birth to 48 months in each sex. This is achieved through analyses of how linear dimensions change with age in each sex using 3D landmarks and semilandmarks located over the cranium (see Fig. 1A-C, Supplementary Fig. S1 and Table S1); (2) to study the ontogenetic allometry of cranial shape in this sample (covariation of the landmark and semilandmark configuration shape with centroid size, after the Gould-Mosimann school^31^) and relate this to how cranial volumes occupied by soft tissues and the nasal cavity (Fig. 1D) change with age. The analyses with respect to aim 2 allow the proportional contribution of volumetric changes to overall cranial growth and development to be estimated, which is important in understanding the factors that drive the development of adult form.

## Results

### Size changes during ontogeny

**Figure 2:**
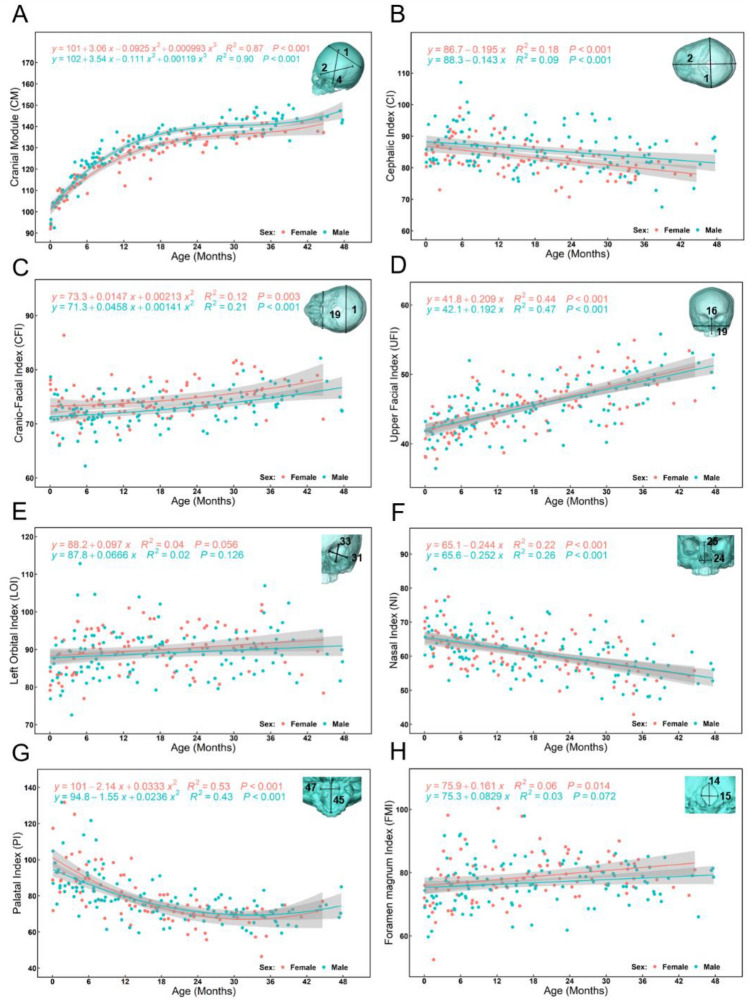
Changes in the cranial module and key skull indices derived from linear measurements in males (blue) and females (red) from birth to 48 months of age: (**A**) Cranial Module (CM = (Maximum cranial length (2) + Maximum cranial breadth (1) + Basion-Bregma height (4))/3). (**B**) Cephalic Index (CI = Maximum cranial breadth (1)/Maximum cranial length (2) × 100). (**C**) Cranio-Facial Index (CFI = Bizygomatic breadth (19)/Maximum cranial breadth (1) × 100). (**D**) Upper Facial Index (UFI = Upper facial height (16)/Bizygomatic breadth (19) × 100). (**E**) Left Orbital Index (LOI = Orbital height (Left, 33)/Orbital breadth (Left, 31) × 100). (**F**) Nasal Index (NI = Nasal breadth (24)/Nasal height (26) × 100). (**G**) Palatal Index (PI = Internal Palatal width (47)/Maximum palatal length (45) × 100). (**H**) Foramen Magnum Index (FMI = Foramen magnum breadth (15)/Foramen magnum length (14) × 100). All regression curves are reported with 95% confidence intervals. Note that the linear dimensions (black) used to calculate each module or index are numbered and shown on the inset transparent skull models (cyan) in the top-right corner of each figure. The detailed definitions of the linear dimensions are presented in Supplementary Table S2.

Figure 2 and Supplementary Table S2 summarise the linear measurements, volumes and indices computed for all individuals and 9 age groups from 0 to 4 years of age (n = 217). The range of natural logarithm of centroid size (Ln(CS)) within each of the 9 age groups for each sex was computed and is presented in Figure S2A. How the cranial module (CM) and calculated indices vary with age is presented in Fig. 2. CM shows the strongest relationship with age, as assessed by a regression analysis (slope = 0.86, R^2^ = 0.86, *p*-value < 0.001). The upper facial index (UFI) also linearly increases with age, in both sexes, but this relationship is weaker than that of CM with age (slope = 1.67, R^2^ = 0.46, *p*-value < 0.001). Other measured indices show non-linear associations with age. For example, palatal index (PI) decreases from birth to about 30 months of age and then gradually increases (Fig. 2G).

**Figure 3:**
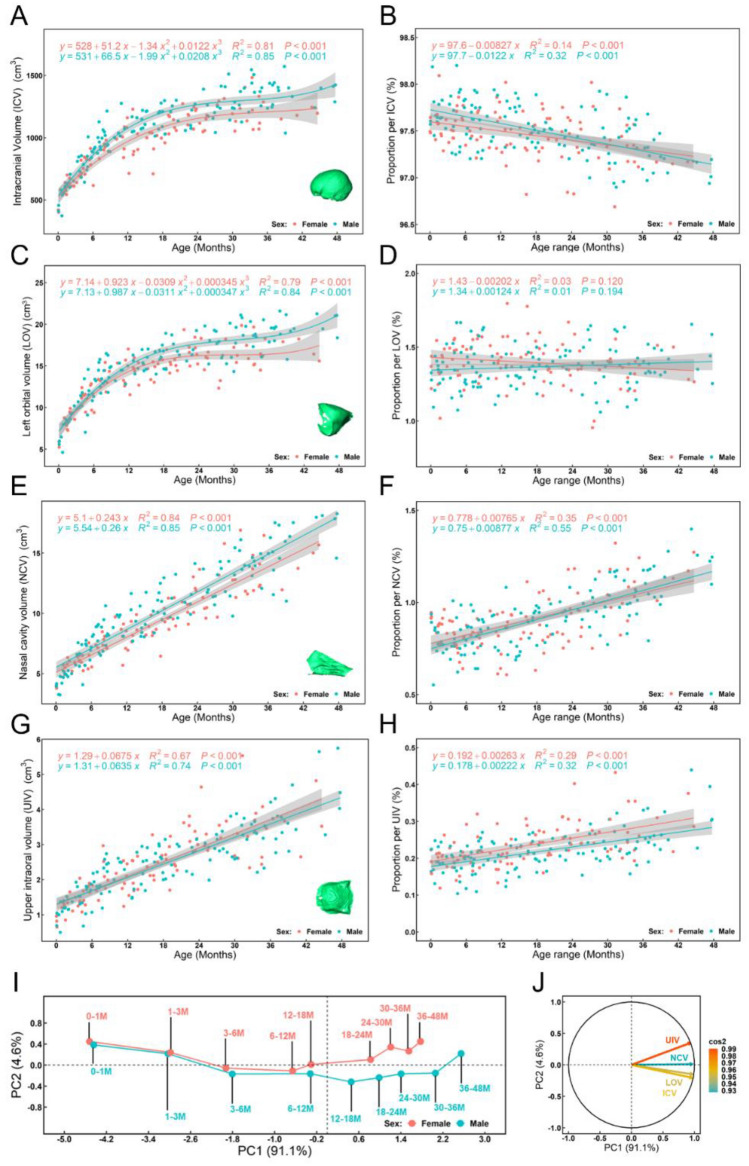
Changes in four cranial volumes from birth to 48 months of age: (Left column) (**A**) Intracranial volume (ICV). (**C**) Volume of the left orbit (LOV). (**E**) Volume of the nasal cavity (NCV). (**G**) upper intraoral volume (UIV). All regression curves are reported with 95% confidence intervals; (Right column, **B**, **D**, **F** and **H**) Changes in the proportional contributions of each volume during growth, expressed as a percentage of each volume after cube root transformation to the sum of four volumes after cube root transformation per individual. (**I**) The first two PCs (PC1 = 91.1% of total variance, PC2 = 4.6%) computed for all 217 individuals, using the matrix of four cranial volumes after cube root transformation (CVols), only the means of the 9 age groups in each sex are plotted (**J**) Loading plot for PC1 and PC2 showing the loadings (squared cosines) of each cranial volume (UIV in orange, NCV in light-blue, LOV in brown and ICV in yellow) on these PCs. Note that there is no significant difference between the volumes of left and right orbits from 0 to 48 months of age (see Supplementary Fig. S5 for details).

Volumetric changes during cranial growth are described in Fig. 3. Intracranial volume (ICV) and left orbital volume (LOV), follow similar patterns of growth (Fig. 3A,C). Both show a rapid increase in the first 18 months of life that substantially slows from 18 to 40 months. Nasal cavity volume (NCV) and upper intraoral volume (UIV) increase linearly with age, at different rates, up to 48 months (Fig. 3E,G). The proportional contribution (expressed as a percentage in Fig. 3B,D,F,H) of each volume to the sum of four volumes after cube root transformation changes over time. Thus, the proportional volume of ICV decreases while those of NCV and UIV both increase with age (Fig. 3B,D,H). How these vary among age groups in each sex is summarised by a principal component analysis (PCA) of the matrix of the four volumes (ICV, LOV, NCV and UIV) from 217 individuals, after cube root transformation (CVols—Fig. 3I). The first two principal components (PCs) account for almost 95% of the total variance (PC1 = 91.1%, PC2 = 4.6%). The first PC reflects overall size variation and allometry (i.e. size related changes in CVols), which is by far the most dominant aspect of variation in this sample. The second PC reflects non-allometric changes in relative proportion among the volumes. The first twelve months are characterised by diminishing PC2 scores and from 12 to 48 months, PC2 scores show a slight increase, most evident in females. Loadings of volumes on PC1 and 2 are shown in diagrammatic form in Fig. 3J. From this it can be seen that NCV is strongly positively loaded (Fig. 3J) on PC1 and that while ICV, UIV and LOV contribute to PC1 scores, they also contribute to scores on PC2, albeit in different ways. The changing proportions of these latter variables contribute to the curvilinearity of the trajectory of growth in PC2. Sexual differences are further explored in Supplementary Table S3, which compares between sexes the regressions of CVols on the natural logarithm of centroid size (Ln(CS)) and age; it can be seen that there are no significant differences except in the regressions of NCV and LOV on size, indicating that these scaling relationships differ between males and females.

### Form (size and shape) changes during ontogeny

**Figure 4:**
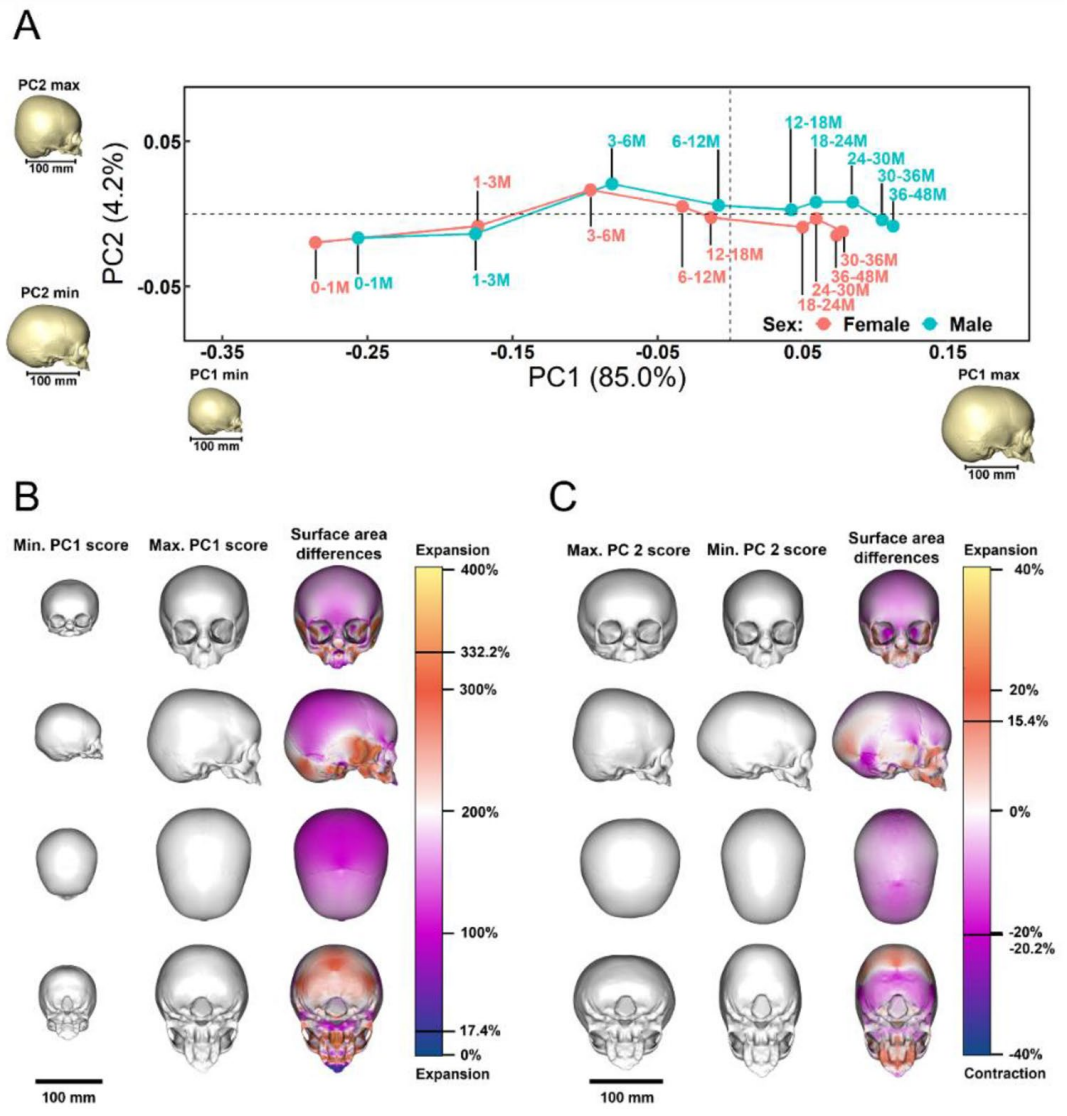
Principal component analysis (PCA) of full landmark and semi-landmark configurations: Form space analysis (shape variables plus the natural logarithm of centroid size (Ln(CS)) of the full sample. (**A**) The first two PCs (PC1 = 85.0% of total variance, PC2 = 4.2%) computed using all 217 individuals with means of each of the 9 age groups then plotted for each sex, (see Supplementary Fig. S2 for the same PCA analysis plotted for the same age groups with combined sex and corresponding Ln(CS) ranges). The inset visualisations of the warped mean cranium were generated by warping the reference skull surface to the extreme negative and positive values of each PC. Colour maps of surface area changes between the cranial surfaces generated by warping the reference skull surface to the extreme negative and positive values of: (**B**) PC1 with the surface area difference ranging from −17.4 to 332.2%. (**C**) PC2 with the surface area difference ranging from −20.2 to 15.4%. Note that the color map (third column) indicates the relative surface area changes from the reference surface (first column) to the target surface (second column). Positive percentages represent relative expansion of specific regions from the reference surface (first column) to the target surface (second column). The two black lines displayed on each right-side scalebar show the limits of surface area expansion and contraction.

An exploratory PCA was carried out using the full landmark and semi-landmark configurations (see Fig. 1B) and Ln(CS) of total 217 samples. The results of PC1 vs. PC2 (a form space analysis, based on the shape variables plus Ln(CS)) plotted using the means of 9 age groups from newborn to 48 months in each sex are shown in Fig. 4. The first two PCs accounted for almost 90% of the total form variance (PC1 = 85.0% of sample variance explained, PC2 = 4.2%—Fig. 4A). Scores on PC1 strongly reflect Ln(CS) (loading = 0.986) and so, age, and the inset warpings of the mean to either extreme of this PC (Fig. 4A,B) indicate that the form differences are strongly reminiscent of growth allometry, with, from left to right, increasing overall size, diminishing relative size of the neurocranium and increasing relative facial size. PC2 accounts for a far smaller proportion of the total variance of the sample including 217 individuals and the inset warpings in Fig. 4A,C, indicate that positive PC2 scores are associated with a flattened occipital and relatively tall vault, with the opposite for negative scores. There is a slight trend towards more positive PC2 values between 3 and 18 months. There is little evidence of sexual dimorphism, except that newborn males appear larger than females and males extend their scores further along PC1, reflecting greater size and accompanying allometric shape differences after approximately 6 months. From the plot of Ln(CS) vs. age group (Supplementary Fig. S3A), it is evident that Ln(CS) increases rapidly in both sexes until the 12^th^ month, and then the rate of increase diminishes. The ranges of Ln(CS) corresponding to 9 age groups were derived from the data used in this plot and how well these relate to age groups was further assessed by performing a PCA of shape of all individuals and then calculating and plotting the means of the 9 age groups and the means of the individuals within the centroid size range of each age group (i.e. including individuals of any age that lie within that size range), while ignoring sex (Supplementary Fig. S3B). We also compared the mean male and female cranial surfaces obtained by a multivariate regression of shape on Ln(CS) on pooled sex data (Supplementary Fig. S3C).

### Comparisons of ontogenetic trajectories between sexes

Table 1 presents the results of a series of multivariate regressions that assess the relationships between cranial shape, cranial form, absolute and relative cranial volumes and Ln(CS) or age, in both sexes. The regressions of shape on Ln(CS) (allometry) or age are significant and both account for approximately 15% of total shape variance within each sex. The permutation test on the angles between sex trajectories is not significant. The regressions of cranial form on Ln(CS) and age explain far greater proportions (84–85%, size; 57–58%, age) of total variance in each sex than the regressions of shape on these variables, because ontogenetic size increase is a major source of variation. However, all these regressions are insignificantly different between sexes. Likewise, the regressions of the cube roots of cranial volumes (CVols) on Ln(CS) and age explain large proportions of total variance (83–87%, size; 69–71%, age) in CVols and are not significantly different between sexes. The regressions of cranial volume proportions after cube root transformation (CPVols) on Ln(CS) or age, like the regressions of shape on these variables, explain a smaller proportion (10–16%, Ln(CS); 24–31%, age) of total variance in proportional volumes but differ significantly (*p*-value < 0.05) between sexes.

**Table 1:** Comparisons between sexes of ontogenetic trajectories from multivariate regressions of cranial shape, form, cube root cranial volumes (CVols-ICV: intracranial volume; NCV: nasal cavity volume; LOV: left orbital volume; UIV: upper intraoral volume) and cranial volume proportions after cube root transformation (CPVols) on Ln(CS) and age in months. *p*-Values are assessed via permutation tests (n = 1000).

Multivariate regression	Females		Males		Ontogenetic vector comparisons	
	R^2^	*p*-Value	R^2^	*p*-Value	Angle (°)	*p*-Value
Shape vs. Ln(CS)	0.15	0.001***	0.14	0.001***	24.1	0.140
Shape vs. age	0.15	0.001***	0.15	0.001***	22.4	0.120
Form vs. Ln(CS)	0.84	0.001***	0.85	0.001***	4.2	0.150
Form vs. age	0.58	0.001***	0.57	0.001***	4.9	0.210
Cube root cranial volumes (CVols) vs. Ln(CS)	0.83	0.001***	0.87	0.001***	3.9	0.060
Cube root cranial volumes (CVols) vs. age	0.69	0.001***	0.71	0.001***	3.6	0.260
Cranial volume proportions after cube root transformation (CPVols) vs. Ln(CS)	0.10	0.001***	0.16	0.001***	31.9	0.027*
Cranial volume proportions after cube root transformation (CPVols) vs. age	0.24	0.001***	0.31	0.001***	19.1	0.040*

Significance: ***p ≤ 0.001, **p ≤ 0.01, *p ≤ 0.05.

### Mean craniofacial shape model

**Figure 5:**
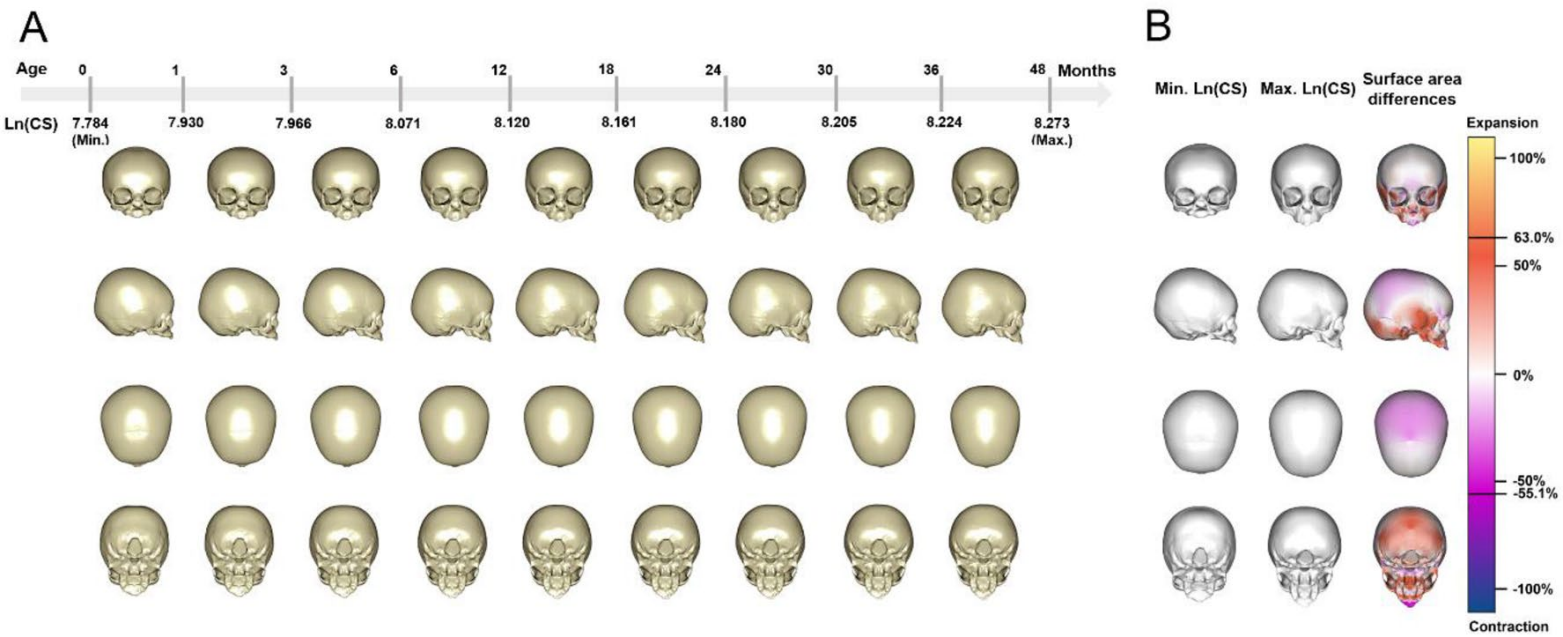
(**A**) The mean cranial surfaces derived by regression of shape on the natural logarithm of centroid size (Ln(CS)) for 9 age groups. Note, sex is combined. The cranial sizes at which these visualisations have been produced are shown along the top scale. Above this, ages are shown, but it should be noted that males and females achieve similar sizes at different ages and so these age estimates are approximate and in reality, differ between sexes, although their allometry is shared (heterochrony). (**B**) Colour maps of surface area changes between the cranial surfaces derived by regression of shape to the minimum and maximum values of the Ln(CS). The range of surface area difference is from −55.1 to 63.0%. Note that the color map (third column) indicates the relative changes in surface area between the reference (first column) and target surfaces (second column). Positive percentages represent relative expansion of specific regions from the reference surface (first column) to the target surface (second column). The two black lines displayed on scalebar show the limits of surface area expansion or contraction.

The findings regarding ontogenetic trajectories allow us to pool males and females to focus on their shared allometric scaling. Using pooled sex data, a multivariate regression of shape on Ln(CS) was carried out. The mean shape with the accompanying template surface mesh was warped along the regression vector to visualise the mean sizes and shapes of crania at 9 age groups with approximately equal size intervals. These visualisations of mean shapes at varying sizes are shown in Fig. 5A. To assess growth changes further, a colour map showing relative changes in shape of cranial regions at minimum and minimum Ln(CS) (see Materials and methods section) is shown in Fig. 5B.

### Interactions between the volumes of intracranial structures and shape during ontogeny

**Figure 6:**
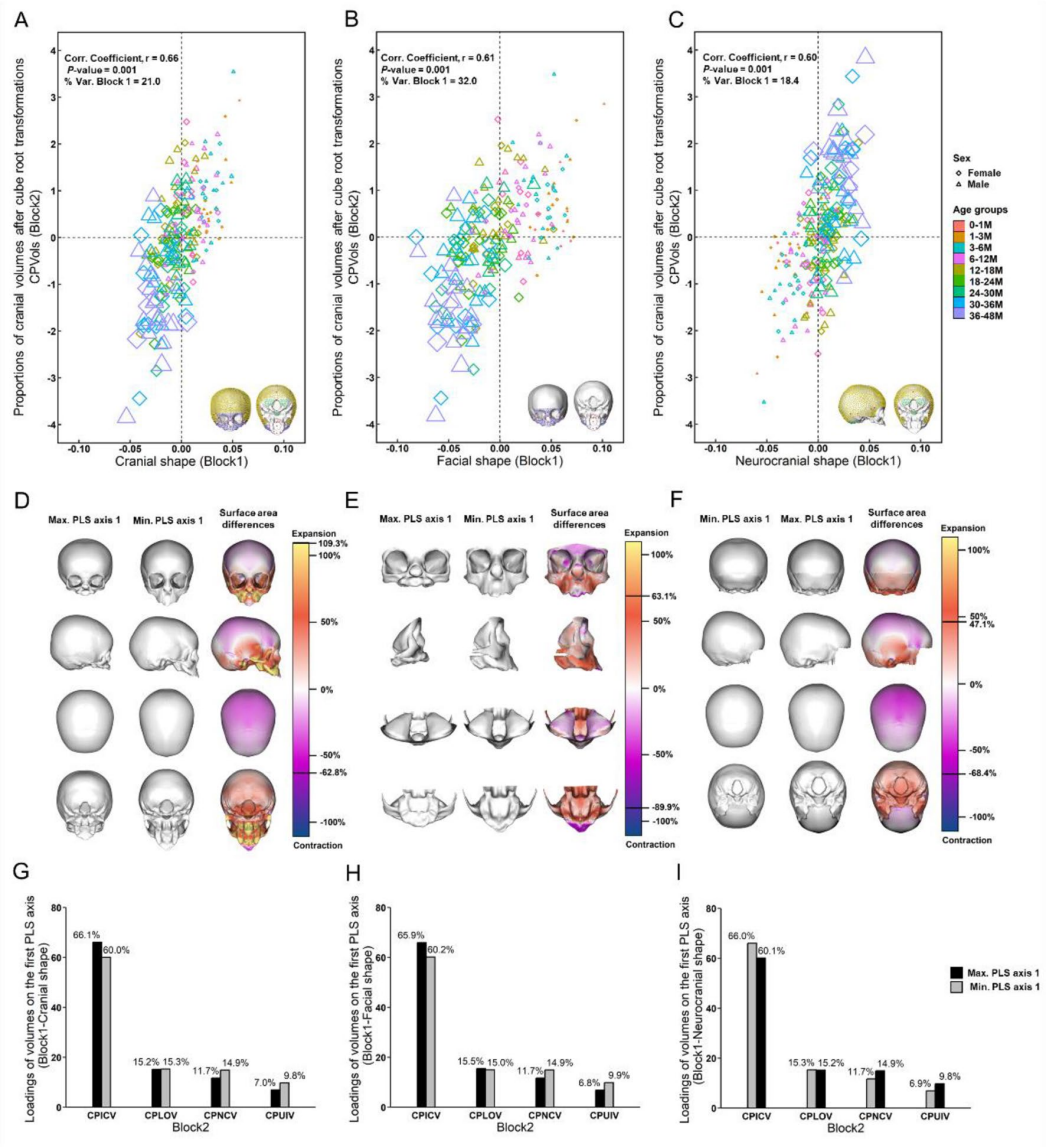
Partial least squares (PLS) analyses between cranial shape, facial shape, and neurocranial shape (Block 1) and proportions of cranial volumes (ICV, LOV, NCV and UIV) after cube root transformation (CPVols—Block2). Plot of PLS axis 1 (Block1) vs. CPVols (Block2): (**A**)–(**C**) Individuals. Diamonds = females, Triangles = males. The marker sizes and colours indicate the age groups of these individuals. Correlations of scores on the first PLS axis (r) and their significance (*p*) are given, together with the percentage of total variance of Block1 explained by its association with Block2 (% Var. Block1). The *p*-value was assessed by running 1000 permutations. The template surface with LMs and SLMs at the bottom of each plot indicates the regions of cranium, face and neurocranium analysed in each plot (see Fig. 1 for details of LMs and SLMs). See Supplementary Fig. 3S3 for the details of the same PLS analyses with means of 9 age groups (in months) and sexes plotted, and also the PLS analyses between cranial form and CPVols. Colour maps of surface area changes between the cranial, facial and neurocranial surfaces at the extremes of PLS axis 1 scores are reported in the third row. The ranges of surface area difference are: (**D**) from −62.8 to 109.3%. (**E**) from −63.1 to 89.9%. (**F**) from −47.1 to 68.4%. See captions in Fig. 5 for details of color mapping. (**G**)–(**I**) Loadings of the cranial volume proportions after cube root transformation (CPICV, CPLOV, CPNCV and CPUIV—Block2) on the first PLS axis.

The two- block partial least square (PLS) analysis between cranial shape and CPVols indicates in a significant correlation among the first PLS axes of each block (r = 0.66, *p*-value = 0.001—Fig. 6A and Supplementary Table S4) and account for 96.7% of the total covariance between blocks (Supplementary Table S5—4th column). The covariation of cranial shape with CPVols explains 21.0% of the total variance of cranial shape (Fig. 6A). The correlations of facial and neurocranial shapes (from separate PLS analyses using facial or neurocranial landmark configurations alone—see Fig. 1B for details of landmark configurations) with CPVols are equal to 0.61 (*p*-value = 0.001) and 0.60 (*p*-value = 0.001), respectively (Fig. 6B,C and Supplementary Table S4). The percentage of the total variance of each of the two modules (face and neurocranium) explained by their covariation with CPVols is equal to 32.0% and 18.4% (Fig. 6B,C). PLS analysis of cranial form against CVols was also performed, obtaining high values of the correlation coefficient between form and CVols (see Supplementary Information 1 and Supplementary Fig. S3A–C).

From the visualisations of changes in shape of the cranium (Fig. 6D), face (Fig. 6E) and neurocranium (Fig. 6F), the variation in cranial shape along the first PLS axis (Block 1) from positive to negative values is related to cranial development (shape changes during the time). At extreme negative value (oldest/largest individuals—Fig. 6D) the entire facial complex is characterized by an increase in area relative to the neurocranium, particularly the maxilla and nasal region. The temporal bone and zygomatic arches are expanded, while the calvarium is characterized by a relative contraction, and the posterior and inferior parts of the occipital bone (nuchal plane, condylar and basioccipital) are characterized by a relative increase in area (Fig. 6D). When the PLS analysis is limited to the facial region (Fig. 6E), it can be seen that the relative expansion of the maxilla is associated with a slight relative contraction of the supraorbital region, and the anterior portion of the dental arch, while the palatal region and the zygomatic arches are both relatively expanded. When the PLS analysis is limited to the neurocranium (Fig. 6F), the results for this region are highly consistent with those from the PLS analysis of the cranium as a whole.

In Fig. 6G–I how CPVols load on the first PLS axes for the three analyses is shown. The loadings do not change much between PLS analyses of the shape of the cranium as whole and those of the face and neurocranium separately. When analysed individually, the proportional contributions of each of the four craniofacial cavities to the total volume significantly covaries with cranial shape (Supplementary Table S4). ICV, NCV and UIV explain between 19.9 and 23.5% of cranial shape variance while OV explains only 6.8% of cranial shape variance (Supplementary Table S4—4th column). NCV and UIV both explain 31.5% of total shape variance in the face while OV explains 15.7% of total facial shape variance (Supplementary Table S4—4th column). Among the cranial volumes, ICV is most highly correlated with neurocranial shape (r = 0.58, *p*-value = 0.001; Supplementary Table S4).

Finally we compared the aspects of shape variation represented by PLS1 from this analysis of shape and CPVols with the shapes derived at the maximum and minimum Ln(CS) from the multivariate regression of shape on size. This was done to assess the extent to which proportional volumes alone represent allometric shape changes (see Supplementary Information 2 and Supplementary Fig. S4 for details). For both comparisons, cranial regions differ little. These findings indicate that proportional cranial volumes alone can represent allometry with small errors, particularly in the region of the anterior maxilla and palate.

## Discussion

This study examined how the cranium changes in form (size and shape) during the first 48 months of life in each sex and it has assessed the ontogenetic allometry of the volumes occupied by soft tissues and the nasal cavity.

Considering the first aim of the study, in detailing the growth of the cranium between 0 and 48 months through analyses of size and shape variation, some evidence of sexual dimorphism in the regional growth of facial skeleton was found. Regressions of volumes on Ln(CS) or age (Supplementary Table S3) indicate that the growth of LOV and NCV are significantly sexually dimorphic, while no differences were recorded either in overall cranial shape or form between sexes (Table 1). Sex-specific growth of the orbit and nose was also reported in a recent study by Syutkina et al*.*^32^ They observed fluctuations in the development of sexual dimorphism in the mid-facial region: it increases within the first year, fluctuates during the second year and peaks during the third and fourth year. The size and volumetric measurements reported here seem to reflect this trend (Fig. 3C,E). Thus, there is no evidence of sexual dimorphism in the ontogenetic trajectories of overall cranial shape or form over the first 48 months, but differences are found between sexes of the same age in average centroid size and volumes of orbits and nose. Thus, average cranial centroid size differs between males and females, but there is no significant sexual dimorphism of growth or development of overall cranial shape or form in the age range considered in this study. These results are in agreement with previous studies performed on head circumference (size) in children from during the first years of life^33,34^. However, similar studies comparing cranial growth (how size increases over the time) during the first years of life have found no differences between sexes^35^. Indeed, sexual differences in cranial growth become most pronounced after puberty, mainly as a result of growth hypermorphosis in males relative to females^36^.

The second aim of this study concerns ontogenetic allometry, assessed through PCAs of shape and form, multivariate regressions of shape and form on age. By applying a recently published method of assessment of the inner volume of cranial cavities^37^, we were able to measure both absolute and proportional changes in volumes of specific cranial regions and relate changes in these to cranial allometric growth. The cavities of the brain, tongue, eyes and nose were assessed because they are believed to play key roles in driving the morphological changes observed in the growing cranium^24,37–39^. Our study shows the accelerations and decelerations of changes in form during early childhood (0–48 months of age). From 0 to 12 months of age, the cranium undergoes greater changes in form than between 12 and 48 months of age. Principal components analysis of size and shape variation of the cranium (PCA of form, i.e. shape variables plus Ln(CS)—Fig. 4) strongly reflects allometry. The spacing of age groups along the first principal component reflects early rapid growth as is seen for intracranial and orbital volumes. The slowing of growth after 12 months of age is also evident from a slowing in the rate of increase in Ln(CS) (Supplementary Fig. S2A). Shape changes relate more linearly to size than age, suggesting a degree of heterochrony. Every region expands in size, but the growth of the posterior aspect of the cranial vault, the hard palate and posterior zygomatic predominate. From 0 to 48 months of age, more than 80% of the variance in craniofacial form is related to size change (see Table 1—3rd row) but only 15% of shape variance is related to size (see Table 1—1st row). Since most size variation in the sample is due to growth, this indicates that changes in size rather than shape predominate over this period. Considering shape changes with size (allometry), the facial region grows more in height than breadth (upper facial index—Fig. 2D). This relates to the mosaic pattern of growth of the facial skeleton and its associated cavities, which reflect their respective functional demands. The main function of the nasal region^15^ (i.e., breathing, conditioning of the inspired air) is reflected by the pattern of growth of the midface (both in tempo and mode, see Fig. 2). Similarly, the increase in nasal volume drives steady growth of the face during the whole period under consideration (Fig. 3E). At the same time, other volumes, such as the intracranial volume (Fig. 3A) and orbital volume (Fig. 3C) show an initial rapid growth, but this slows considerably after 12 months.

Our results highlight the importance of the rapidly growing brain and, to a lesser extent, eyes as driving forces of neurocranial and upper facial growth in the first 12 months (Fig. 3A,C). Increases in the upper intraoral volume (accommodating growth of the tongue and dentition) and in nasal cavity volume are continuous throughout the first 48 months (Fig. 3E,G). These mainly relate to, and may drive, growth of the face^15^. The difference in growth trajectories between these volumes and the intracranial and orbital volumes underlie the differences in cranial proportions highlighted earlier. Beyond 12 months, brain and eyes continue to grow but at much slower rates. How the volumes of craniofacial regions relate to overall cranial growth and development was examined through PLS analyses (Supplementary Tables S3,S4) of cranial form vs. cranial volumes (Supplementary Fig. S3A–C) or cranial shape vs. cranial volume proportions (Fig. 6). The very strong associations found in the PLS analysis of cranial form vs. cranial volumes highlights the importance of size changes with age. Thus, from Supplementary Table S4, 84.8% of variance in cranial form is explained by its association with cranial volumes. Shape is also significantly associated with the proportions of cranial volumes, but this association only explains 21% of shape variance (Fig. 6A), a very similar proportion to that explained by growth allometry (Fig. 6D). The remaining 79% is not explained by these volumes and the evidence of Fig. 6D is that the aspects of shape variance that are not explained are likely not related to growth or development, but to individual variation. Separate analyses of facial and neurocranial shape vs. CPVols (Fig. 6B,C,E,F) support these conclusions. Future studies are needed to explore this further and to identify other factors that might account for growth allometry. Candidates include muscle forces^26^, masticatory system loading and volumes of other soft tissues. However, from these PLS analyses it is evident that relative expansion of cranial volumes is significantly related to cranial ontogenetic shape changes over the first 48 months. This might simply reflect associations between cranial volumes and size and between cranial shape and size or it may reflect the direct effects of expansion of volumes. This can be assessed in future studies simulating the mechanical interactions of volumes with adjacent skeletal structures. Thus, these findings with respect to the association between cranial volumes and changes in cranial size and shape provide new insights into growth interactions and new data relevant to future mechanical modelling studies aiming to predict growth based on the expansions and actions of developing soft tissues^29,30^.

Beyond these factors, biological and genetic variations can also affect growth allometry. For example, the rates and timings of suture closures can impact craniofacial shape. The metopic suture is the only significant one which is fully fused at around 9 months of age while the others (i.e. sagittal, coronal, lambdoid and squamosal sutures) remain patent during infancy^40^. In craniosynostosis^10^, early fusion of cranial sutures leads to abnormal craniofacial growth and development.

This study has several limitations: (1) the CTs were obtained from a craniofacial unit in France and may not well represent the global population^41^, and the samples were not equally distributed throughout the studied age range (e.g. there are 88 individuals in the first year of life while there are only 21 individuals in the group of 36 to 48 months of age). Furthermore, because of the limitations of data availability, sexes are unbalanced with an excess of males. These aspects may have affected the findings. As more CT data become available, future studies should address these deficiencies with more even sampling of ages and sexes of more geographically diverse populations. (2) the 88 anatomical landmarks for linear measurements for each sample were manually placed, but by the same person to minimise inter-observer error. 10 LMs are identified at the sutures between cranial bones. When the sutures/bones were not yet fully formed, we referred to all reconstructed skull models up to three months older than the specimen being landmarked to estimate the shapes of unformed bones, and identified the sutures to be formed on the relevant CT slices. Most of the LMs are based on general anatomical features and the literature. As there is an inevitable error in landmarking^42^, author defined LMs were reviewed to ensure landmark precision and repeatability. (3) analyses related to cranial and facial sutures were not involved in this study. The ‘gaps’ on the reconstructed skull models caused by the unfused sutures (e.g. anterior fontanelle) were manually filled before semi-landmarking; (4) the semi-landmarks were only located on the external surface of each cranium and so the surface warping of unlandmarked regions is unlikely to be accurate (e.g. anterior part of skull base); (5) the detailed analysis of directional (i.e. changes in shape and shifts in location relative to other parts of the cranium) changes of craniometric volumes during ontogeny is not considered and will be addressed in our further work. (6) we examined the relationships between only four cranial volumes and cranial shape or form in this study, but the contributions of other volumes e.g. frontal and maxillary sinuses^37^ may also be important and should be investigated in further work.

In summary, this study characterises and models neonatal skull growth, showing that it is allometric, that changes in size predominate and that changes in shape are associated with changes in the sizes and relative proportions of internal organs and capsules of the craniofacial system.

## Materials and methods

### Sample and image processing

Computed tomography (CT) stacks of the heads of 217 individuals (94 females and 123 males) from 0 to 48 months of age were used in this study: 88 individuals (39 females and 49 males) from 0 to 12 months of age, 60 individuals (30 females and 30 males) from 12 to 24 months of age, 48 individuals (21 females and 27 males) from 24 to 36 months of age, and 21 individuals (4 females and 17 males) from 36 to 48 months of age (see Supplementary Fig. S6 for more details about sample distribution throughout the studied age range). The actual age of individuals in days when scanned was obtained from their reported dates of birth and registered CT scan records. To specify age ranges, all age values are given in months, to two decimal places (with 1 month equal to 30.41 days). The whole age range of female individuals is from 1 to 1357 days (≈ 44.62 months), and that for male individuals is from 2 to 1451 days (≈ 47.71 months). These individuals were born between 2008 and 2018, scanned for clinical purposes to investigate minor trauma, and individuals with bone lesions and seizures were excluded. The scans were subsequently made available for this study.

All CT data were anonymised and provided by the Necker-Enfants Malades University Hospital in Paris where all data collection and analysis (methods) were performed in accordance with the relevant guidelines and regulations. The full ethical protocol for undertaking this study was approved by the institutional review board and committee of the Necker – Enfants Malades University Hospital (study No. 2018RK18). Informed consent was granted from the patient’s parent or guardian. The other details of these individuals, i.e., ethnicity and body weight, were not available for this study. CT images were reconstructed in Avizo Lite software (FEI V9.2., Thermo Fisher Scientific, Mass, USA). The CT stacks were semiautomatically segmented with manual refinement to isolate the cranium from the soft tissues and mandible before making 3-dimensional cranial reconstructions.

### Landmarking

88 anatomical landmarks (LM) were located on each cranium. Some landmarks are registration dependent and so all crania were landmarked after orientation to the Frankfort horizontal. See Fig. 1, and Supplementary Table S1 for details of each LM^20,43–47^. These LMs were used to compute linear measurements and to guide the placement and sliding of surface semilandmarks^48,49^, as shown in Fig. 1. The landmarking process was performed in the EVAN Toolbox (v1.75, EVAN-Society)^50^ and Avizo Lite software (FEI V9.2., Thermo Fisher Scientific, Mass, USA), and subsequent semi-landmarking was carried in R (see below for details). The majority of LMs were placed on surface features of craniofacial bones or at their sutural junctions. However, because the bones and sutures are not fully developed in the early postnatal period, the positions of 10 LMs (including No. 48-*Bregma*, 49- *Lambda*, 52&53-*Sphenion*, 54&55-*Krotaphion*, 58&59-*Entomion*, and 60&61-*Asterion*), which are defined at the intersections of cranial sutures and/or bones, were estimated based on local anatomical features of reconstructed skull models and suture positions identified from CT scans.

### Measurements

The three-dimensional coordinates of the LMs were used to calculate 47 linear measurements describing the dimensions of cranial components (Fig. 1C, see Supplementary Table S2 for detailed definitions of each measure)^20,23,43,44,51–53^. Additionally, a classical measure of head size based on the mean of the length, breadth, and auricular height of the braincase^54–56^, and termed the ‘cranial module’ (CM) was calculated together with 7 cranial indices. The definitions of cranial module and indices measured in this study are detailed in Fig. 2 and Supplementary Table S2C. To characterise the size changes of key internal structures and organs of the craniofacial system, intracranial volume (ICV), orbital volume (OV), nasal cavity volume (NCV) and upper intraoral volume (UIV) were measured (Fig. 1D). These volumetric measurements were extracted from cranial surface scans and taken using the R package: *Arothron* and the functions *Icex* and *Icv*^37,57^. The raw volumetric measurements were cube root transformed to obtain a normal distribution. Further, the proportional contributions of (cube root transformed) volumetric measurements to the sum of these measurements were calculated for subsequent analyses of the association between age related changes in their relative contributions and the development of craniofacial form. The anatomical boundaries used to measure these volumes are shown in Supplementary Fig. S1. Orbital volume was calculated for only the left side. In a number of cases, throughout the studied age range (0 to 4 years), both sides were measured, and these show a high degree of concordance, reflecting symmetry (Supplementary Fig. S5).

### Surface semi-landmark configuration

While LMs have precisely defined locations, they have limited applicability over smooth surfaces because few identifiable homologies can be recognised. For this reason, surfaces were parameterised using semi-landmarks (SLMs), which lie between the LMs and whose location is determined by the LMs. Based on Procrustes distances calculated using the LMs, the cranium most similar to the mean, individual F_464 (female, 464 days after birth), was chosen as the template to seed semi-landmarking. The 3D external surface mesh was extracted from the reference skull using the R function *out.inn.mesh*^58^. The template SLMs were distributed over patches, to build the complete template. On one side of the skull, three surface mesh patches representing the midfacial, calvarial and lower occipital regions were labelled in Geomagic Wrap (v2017, 3D Systems Inc., Morrisville, North Carolina—see Supplementary Fig. S1 for detailed boundaries). To capture the anatomical features at small scales through the studied age range and get detailed shape or form visualizations^59,60^, a denser set of SLMs were determined and then sited on each: 100 on the midfacial patch, 1000 on the calvarial patch and 52 on the lower occipital. The set of surface SLMs was mirrored and projected to the other side of the skull. This resulted in 2304 approximately evenly distributed surface SLMs over the template, including 200 SLMs over the mid-face and 2104 SLMs over the neurocranium (Fig. 1B). The R packages *Arothron*^57^, and *Morpho*^61^ were used to project them to, and slide them over each target cranium. The sliding algorithm minimised the bending energy^62,63^ of a triplet of thin plate splines. This minimisation of bending energy is intended to minimise error (variance of the sample) due to differences in SLMs location. This is, by now, the standard approach in morphometric studies of skeletal form^61^. Our newly published experiments^60,64^ indicate that it achieves similar results to those from the non-iterative closest points (NICP) method for point cloud registration^65^, commonly applied in computer vision. However, neither approach can ensure homology of SLMs^66,67^. Instead, both are compromises that minimise the extent to which ‘errors’ in SLM location inflate apparent differences (as assessed by e.g. Procrustes distances, see below).

### Statistical analyses

#### Cranial volumes

To investigate how the four cranial volumes (ICV, LOV, NCV and UIV) change throughout the first 48 months, they were plotted against age and compared between sexes. To assess how these change collectively, a PCA was undertaken of the matrix of four cranial volumes of 217 individuals after cube root transformation (CVols) and of their proportional contributions to the total of these volumes after cube root transformation (CPVols), using the basic R package *stats*. The loadings of each of the four volumes on principal components (PCs 1 and 2) were reported.

#### Cranial form (size and shape) variation with age and sex

Generalized Procrustes Analysis (GPA) was carried out of the entire sample, using the set of 86 LMs (the paired LMs for Euryon were excluded during superimposition as their positions change with skull shape) and 2304 SLMs to compute shape variables and centroid sizes for subsequent statistical analyses. The resulting matrix of shape variables comprises the coordinates of the LMs and SLMs after GPA. To assess growth (changes in size over time), the natural logarithm of centroid size (Ln(CS)) was plotted against and regressed on age in months. The significance of the angle (computed as the dot product) between multivariate vectors^68^ was tested using a permutation test (1000 permutations). The matrix of shape variables (shape matrix) augmented with a column comprising the Ln(CS) (here called the form matrix) was used to calculate the mean form of 9 pooled age groups (1 group between 0–1 month, 1 group between 2–3 months, 1 group per 3 months between 3 and 12 months, 1 group per 6 months between 12 and 36 months and 1 group comprising individuals between 36 and 48 months). Note that the age intervals were determined by considering the uneven distribution of original samples (see Supplementary Fig. S6) and the rapid growth rate in the first year of life. The major modes of variation in form among these groups were investigated through PCA of the matrix of shape variables augmented with a column comprising the Ln(CS) (PCA of form). To simplify visualisation, the means of each group are plotted. Allometry (size related changes in shape) and development (age related changes in shape) as well as age and size related changes in form were compared between sexes through multivariate regression of the shape or form matrices on Ln(CS) or age.

To investigate how cranial shape or cranial form covaries with cranial volumes or cranial volume proportions, two-block partial least squares analyses (PLS)^69–71^ were carried out. For analyses of shape and relative proportions of cranial volumes, the two blocks of variables comprise either cranial shape, facial shape or neurocranial shape (Block 1) vs. (Block 2) the matrix of the proportional contributions of each volume to the total of all measured intracranial volumes after cube root transformation (CPVols). This relates cranial shape variation (cranial proportions) to variation in relative proportions of cranial volumes between 0 and 4 years. For the PLS analyses of form vs cranial volumes, the two blocks of variables comprise cranial form (Block 1) vs. (Block 2) the cube root transformed cranial volumes (CVols). This relates size and shape (form) variation of the cranium to variation in cranial volumes between 0 and 4 years. These PLS analyses used different landmark configurations (2304 SLMs for the whole cranium, 200 SLMs for mid-face and 2104 SLMs for neurocranium—see Fig. 1B) from all 217 specimens, and were reported by plotting individuals and the means of the 9 pooled age groups described above for each sex.

#### Visualisation

To relate the modes of variation represented by vectors of interest (principal components, multivariate regression vectors, or PLS vectors) to morphology, the overall mean cranial shape or form was warped along the vector to the score of interest. This generated the set of LMs and SLMs represented by that score. To facilitate visualisation, the surface mesh of the template (see above) was then warped to fit this set of LMs and SLMs using the R package *Morpho*^61^. In shape analyses, size was restored according to the Ln(CS) associated with that score directly from the multivariate regression. To visualise changes in shape or form between two meshes, one was selected as the reference (e.g. the younger) and the other (e.g. the older) as the target. To avoid registration issues^72^ visualisation of differences focussed on local changes in area of the surface mesh, producing a colour map indicating regions of expansion and contraction^60,68^. This is an incomplete visualisation of differences in form of surface meshes, in that omits information about vectors of deformation. However, this information is evident from comparisons of reference and surface mesh form. More complete, registration free, visualisations of deformation are possible using transformation grids computed from thin plate splines^62^ or via visualisation of surface ‘strains’^73^, but these are less easy to present in print and interpret in 3D. To highlight the localised changes in skull surface area between reference and target (e.g. growth), the differences in area of each corresponding triangle between the reference and target meshes were colour mapped onto the target mesh^57^. Note no SLMs were placed on the internal surfaces of the cranium (including the orbital floors), zygomatic arches, or the anterior regions of the base of the skull, and so their apparent deformations in warped surfaces and the colour mapping should be treated as approximations.

### Ethical approval

Ethical approval was obtained for this study from Necker – Enfants Malades University Hospital under №2018RK18.

## Supplementary Information

Supplementary Information.

## Data Availability

All data generated or analysed during this study are included in the manuscript and supplementary tables and figures.

## References

[1] Scott JH (1954). The growth of the human face. Proc. R. Soc. Med..

[2] Moss ML, Young RW (1960). A functional approach to craniology. Am. J. Phys. Anthropol..

[3] Enlow DH (1966). A morphogenetic analysis of facial growth. Am. J. Orthod..

[4] Dekaban AS (1977). Tables of cranial and orbital measurements, cranial volume, and derived indexes in males and females from 7 days to 20 years of age. Ann Neurol..

[5] Enlow DH, Hans MG (1996). Essentials of Facial Growth.

[6] Lieberman DE, Pearson OM, Mowbray KM (2000). Basicranial influence on overall cranial shape. J. Hum. Evol..

[7] O’Higgins P, Cohen MJ (2000). Development, Growth, and Evolution: IMPLICATIONS for the Study of the Hominid Skeleton.

[8] Morriss-Kay GM, Wilkie AOM (2005). Growth of the normal skull vault and its alteration in craniosynostosis: Insights from human genetics and experimental studies. J. Anat..

[9] Bastir M (2010). Effects of brain and facial size on basicranial form in human and primate evolution. J. Hum. Evol..

[10] Richtsmeier JT, Flaherty K (2013). Hand in glove: Brain and skull in development and dysmorphogenesis. Acta Neuropathol..

[11] Jin S-W, Sim K-B, Kim S-D (2016). Development and growth of the normal cranial vault: An embryologic review. J. Korean Neurosurg. Soc..

[12] O’Sullivan E (2021). The 3D skull 0–4 years: A validated, generative, statistical shape model. Bone Rep..

[13] Johnson D, Wilkie AOM (2011). Craniosynostosis. Eur. J. Hum. Genet..

[14] Mathijssen IMJ (2015). Guideline for care of patients with the diagnoses of craniosynostosis: Working group on craniosynostosis. J. Craniofac. Surg..

[15] Landi F (2021). The role of the nasal region in craniofacial growth: An investigation using path analysis. Anat. Rec..

[16] Ferrario VF, Sforza C, Poggio CE, Schmitz JH (1999). Soft-tissue facial morphometry from 6 years to adulthood: A three-dimensional growth study using a new modeling. Plast. Reconst. Surg..

[17] Vidarsdottir US, O’Higgins P, Stringer C (2002). A geometric morphometric study of regional differences in the ontogeny of the modern human facial skeleton. J. Anat..

[18] Bastir M, O’Higgins P, Rosas A (2007). Facial ontogeny in Neanderthals and modern humans. Proc. R. Soc. B..

[19] Krimmel M (2015). Three-dimensional normal facial growth from birth to the age of 7 years. Plast. Reconstr..

[20] Evteev A, Anikin A, Satanin L (2018). Midfacial growth patterns in males from newborn to 5 years old based on computed tomography. Am. J. Hum. Biol..

[21] Delye H, Clijmans T, Mommaerts MY, Sloten JV, Goffin J (2015). Creating a normative database of age-specific 3D geometrical data, bone density, and bone thickness of the developing skull: A pilot study. J. Neurosurg. Pediatr..

[22] Li Z (2015). A statistical skull geometry model for children 0–3 years old. PLoS ONE.

[23] Libby J (2017). Modelling human skull growth: A validated computational model. J. R. Soc. Interface.

[24] Butaric LN, McCarthy RC, Broadfield DC (2010). A preliminary 3D computed tomography study of the human maxillary sinus and nasal cavity. Am. J. Phys. Anthropol..

[25] Goergen MJ, Holton NE, Grünheid T (2017). Morphological interaction between the nasal septum and nasofacial skeleton during human ontogeny. J. Anat..

[26] Toro-Ibacache V, Muñoz VZ, O’Higgins P (2016). The relationship between skull morphology, masticatory muscle force and cranial skeletal deformation during biting. Ann. Anat..

[27] Lieberman DE, Ross CF, Ravosa MJ (2000). The primate cranial base: Ontogeny, function, and integration. Am. J. Phys. Anthropol..

[28] Galiay L (2021). Management of sagittal craniosynostosis: Morphological comparison of 8 surgical techniques. Br. J. Oral Maxillofac. Surg..

[29] Cross C (2021). Predicting and comparing three corrective techniques for sagittal craniosynostosis. Sci. Rep..

[30] Cross C (2022). A computational framework to predict calvarial growth: Optimising management of sagittal craniosynostosis. Front. Bioeng. Biotechnol..

[31] Klingenberg CP (2016). Size, shape, and form: Concepts of allometry in geometric morphometrics. Dev. Genes Evol..

[32] Syutkina T, Anikin A, Satanin L, Evteev A (2023). Sexual dimorphism in human midfacial growth patterns from newborn to 5 years old based on computed tomography. J. Anat..

[33] Broere-Brown ZA (2016). Sex-specific differences in fetal and infant growth patterns: A prospective population-based cohort study. Biol. Sex Differ..

[34] Joffe TH (2005). Fetal and infant head circumference sexual dimorphism in primates. Am. J. Phys. Anthropol..

[35] Bergerat M (2021). Head circumference from birth to five years in France: New national reference charts and comparison to WHO standards. The Lancet Region Health – Europe.

[36] Bulygina E, Mitteroecker P, Aiello L (2006). Ontogeny of facial dimorphism and patterns of individual development within one human population. Am. J. Phys. Anthropol..

[37] Buzi C (2023). Icex : Advances in the automatic extraction and volume calculation of cranial cavities. J. Anat..

[38] Sgouros S, Goldin JH, Hockley AD, Wake MJC, Natarajan K (1999). Intracranial volume change in childhood. J. Neurosurg..

[39] Hansen TI, Brezova V, Eikenes L, Håberg A, Vangberg TR (2015). How does the accuracy of intracranial volume measurements affect normalized brain volumes? Sample size estimates based on 966 subjects from the HUNT MRI cohort. AJNR Am. J. Neuroradiol..

[40] Idriz S, Patel JH, Ameli Renani S, Allan R, Vlahos I (2015). CT of normal developmental and variant anatomy of the pediatric skull: Distinguishing trauma from normality. Radiographics.

[41] Bruner E, Manzi G (2004). Variability in facial size and shape among North and East African human populations. Ital. J. Zool. (Modena).

[42] von Cramon-Taubadel N, Frazier BC, Lahr MM (2007). The problem of assessing landmark error in geometric morphometrics: Theory, methods, and modifications. Am. J. Phys. Anthropol..

[43] Howells WW (1973). Cranial Variation in Man: A Study by Multivariate Analysis of Patterns of Difference Among Recent Human Populations.

[44] Martin R, Knussmann R (1988). Anthropologie: Handbuch der Vergleichended Biologie des Menschen.

[45] Skrzat J, Holiat D, Walocha J (2003). A morphometrical study of the human palatine sutures. Folia Morphol..

[46] Caple J, Stephan CN (2016). A standardized nomenclature for craniofacial and facial anthropometry. Int. J. Legal Med..

[47] Wärmländer SK, Garvin H, Guyomarch P, Petaros A, Sholts SB (2019). Landmark typology in applied morphometrics studies: What’s the point?. Anat. Record.

[48] Gunz P, Mitteroecker P, Bookstein FL, Slice DE (2005). Semilandmarks in three dimensions. Modern Morphometrics in Physical Anthropology.

[49] Gunz P, Mitteroecker P (2013). Semilandmarks: a method for quantifying curves and surfaces. Hystrix It. J. Mamm..

[50] O’Higgins P, Jones N (1998). Facial growth in Cercocebus torquatus: An application of three-dimensional geometric morphometric techniques to the study of morphological variation. J. Anat..

[51] Gruber P, Henneberg M, Böni T, Rühli FJ (2009). Variability of human foramen magnum size. Anat. Rec..

[52] Lesciotto KM, Cabo LL, Garvin HM (2016). A morphometric analysis of prognathism and evaluation of the gnathic index in modern humans. Homo.

[53] Nikolova S, Toneva D, Georgiev I (2017). A case of bipartite zygomatic bone. Eur. J. Forensic Sci..

[54] Weidenreich F (1941). The brain and its role in the phylogenetic transformation of the human skull. Trans. Am. Philos. Soc..

[55] Henneberg M (1988). Decrease of human skull size in the Holocene. Hum. Biol..

[56] Pérez-Claros JA, Jiménez-Arenas JM, Palmqvist P (2015). Neurocranium versus face: A morphometric approach with classical anthropometric variables for characterizing patterns of cranial integration in extant hominoids and extinct hominins. PLoS ONE.

[57] Profico A (2021). Arothron: An R package for geometric morphometric methods and virtual anthropology applications. Am. J. Phys. Anthropol..

[58] Profico A (2018). Reproducing the internal and external anatomy of fossil bones: Two new automatic digital tools. Am. J. Phys. Anthropol..

[59] Mitteroecker P, Schaefer K (2022). Thirty years of geometric morphometrics: Achievements, challenges, and the ongoing quest for biological meaningfulness. Am. J. Biol. Anthropol..

[60] Shui W, Profico A, O’Higgins P (2023). A comparison of semilandmarking approaches in the visualisation of shape differences. Animals.

[61] Schlager S, Zheng G, Li S, Szekely G (2017). Morpho and Rvcg—shape Analysis in R. Statistical Shape and Deformation Analysis.

[62] Bookstein FL (1989). Principal warps: Thin-plate splines and the decomposition of deformations. IEEE Trans. Pattern Anal. Machine Intell..

[63] Mitteroecker P, Gunz P, Windhager S, Schaefer K (2013). A brief review of shape, form, and allometry in geometric morphometrics, with applications to human facial morphology. Hystrix, Ital. J. Mammal..

[64] Shui W, Profico A, O’Higgins P (2023). A comparison of semilandmarking approaches in the analysis of size and shape. Animals.

[65] Serafin, J., & Grisetti, G. NICP: Dense normal based point cloud registration. In *2015 IEEE/RSJ International Conference on Intelligent Robots and Systems (IROS)* 742–749 (2015).

[66] Oxnard C, O’Higgins P (2009). Biology clearly needs morphometrics. Does morphometrics need biology ?. Biol. Theory.

[67] Cardini A (2020). Less tautology, more biology? A comment on “high-density” morphometrics. Zoomorphology.

[68] Smith OAM (2021). 3D Modeling of craniofacial ontogeny and sexual dimorphism in children. Anat. Rec..

[69] Rohlf FJ, Corti M (2000). Use of two-block partial least-squares to study covariation in shape. Syst. Biol..

[70] Bastir M, Rosas A (2016). Cranial base topology and basic trends in the facial evolution of homo. J. Hum. Evol..

[71] Katsube M (2019). Critical growth processes for the midfacial morphogenesis in the early prenatal period. Cleft Palate Craniofac. J..

[72] Moyers RE, Bookstein FL (1979). The inappropriateness of conventional cephalometrics. Am. J. Orthod..

[73] Piras P (2020). Current options for visualization of local deformation in modern shape analysis applied to paleobiological case studies. Front. Earth Sci..

