# Supplementary material for: Normal human craniofacial growth and development from 0 to 4 years
Source: Sci Rep. 2023 Jun 14;13:9641. doi: 10.1038/s41598-023-36646-8 (PMC10267183; doi:10.1038/s41598-023-36646-8)
Supplement: Supplementary file 1 — Supplementary Information. [file 41598_2023_36646_MOESM1_ESM.docx]

**Normal human craniofacial growth and development from 0-4 years**

Ce Liang^1^, Antonio Profico^2^, Costantino Buzi^3,4^, Roman H Khonsari^5^, David Johnson^6^, Paul O’Higgins^7,8^, Mehran Moazen^1*^

^1^Department of Mechanical Engineering, University College London, London, UK

^2^Department of Biology, University of Pisa, Pisa, Italy

^3^Institut Català de Paleoecologia Humana i Evolució Social (IPHES-CERCA), Tarragona, Spain

^4^Departament d’Història i Història de l’Art, Universitat Rovira i Virgili, Tarragona, Spain

^5^Department of Maxillofacial Surgery and Plastic Surgery, Necker – Enfants Malades Hospital, Assistance Publique – Hôpitaux de Paris, Paris, France

^6^Oxford Craniofacial Unit, Oxford University Hospital, Oxford, United Kingdom

^7^PalaeoHub, Department of Archaeology, University of York, York, UK
^8^Hull York Medical School, University of York, York, UK

*Corresponding author: [M.Moazen@ucl.ac.uk](mailto:M.Moazen@ucl.ac.uk)

**Keywords:** craniofacial growth, ontogeny, allometry, sexual dimorphism, geometric morphometrics

**Supplementary Information 1**

**Interactions between the volumes of intracranial structures and form during ontogeny**

The PLS analysis of cranial form against CVols (Supplementary Fig. S3A-C) indicates a significant correlation (r = 0.99, p-value = 0.001 – Supplementary Fig S3A and Table S4) between the first PLS axes of these two blocks. PLS1 explains nearly 84.8% of form variance (Supplementary Fig S3A and Table S4) and 99.9% of the covariance between blocks (Supplementary Table S5). Similar results are obtained when PLS is carried out between cranial form and any single CVol, Thus, the correlation between PLS1 scores from the PLS of intracranial volume alone against cranial form yields identical results to that of the PLS analysis using all CVols (r = 0.99, p-value = 0.001, explained proportion of variance in form = 84.8% - Supplementary Table S4). Upper intra oral volume alone explains the least proportion of cranial form variance (84.7%), and this is also a highly significant result (r = 0.85, p-value = 0.001 - Supplementary Table S4). The other CVols result in correlations and explained variances that are intermediate. Since form relates to both the size and shape of the cranium, these high values of the correlation coefficient and of explained proportions of total variance reflect both size and shape covariance with CVols. The individual CVols all reflect general size increase and so are highly correlated and produce similar results.

**Supplementary Information 2**

**Comparisons between the cranial shapes derived from PLS of shape and proportions of cranial volumes after cue root transformation (CPVols) and multivariate regression of shape on size (Ln(CS))**

We compared the aspects of shape variation represented by PLS1 from this analysis of shape and CPvols with the shapes derived at the maximum and minimum Ln(CS) from the multivariate regression of shape on size. This was done to assess the extent to which proportional volumes alone are associated with allometric cranial shape changes. The results are presented in Supplementary Fig. S4 from which it can be seen that the major differences in warped shape are found in the anterior maxilla and palate. The shape of the youngest/smallest cranium derived from PLS1 (max. PLS1 score - Supplementary Fig. S4A) has a relatively smaller anterior maxilla and palate than the the smallest cranium derived from multivariate regression (min. Ln(CS) - Supplementary Fig. S4A). These regions are relatively larger in the shape of the largest/oldest cranium derived from PLS1 (min. PLS1 score - Supplementary Fig. S4B) than in the largest cranium derived from multivariate regression (max. Ln(CS) - Supplementary Fig. S4B). Elsewhere, for both comparisons cranial regions differ little. These findings indicate that proportional cranial volumes alone can represent allometry with small errors, particularly in the region of the anterior maxilla and palate.

**Supplementary Materials**

**Table S1.** Anatomical landmarks used in the present study including abbreviations and definitions

| **No.** | **3D Notation** | **Landmark** | **Definition** |
| --- | --- | --- | --- |
| **Midline** |  |  |  |
| 1 | g | Glabella | Most anterior point on the lower edge of the frontal bone in the midsagittal plane, on the brow ridge and above the nasofrontal suture. |
| 2 | n | Nasion | The point of intersection of the nasofrontal suture in the midsagittal plane. |
| 3 | rhi | Rhinion | Most caudal (end) point on the internasal suture, between the paired nasal bones. |
| 4 | ns | Nasospinale | The point of intersection of the inferior margin of the nasal aperture with the midsagittal plane. |
| 5 | pr | Prosthion | Midline point on the maxillary alveolar arch between the upper central incisors. |
| 48 | b | Bregma | Most posterior point on the border of the frontal bone in the midsagittal plane, at the intersection of the coronal and sagittal sutures. |
| 49 | l | Lambda | Midline point on the apex of the occipital bone, at the intersection of the sagittal and lambdoid sutures. |
| 50 | op | Opisthocranion | Most posterior point on the occipital bone in the midsagittal plane, between the *Glabella* and *Inion*. |
| 51 | i | Inion | Midline point on the bony prominence of the occipital bone between the apices of the superior nuchal lines. |
| 62 | cpp^a^ | Cribriform plate posterior | Most posterior point on the cribriform plate of the ethmoid in the midsagittal plane, where the rear edge of the plate meets the most anterior part of sphenoid bone. |
| 63 | ol | Orale | Most inferior point of the maxillary symphysis in the midsagittal plane. |
| 64 | sr^b^ | Staurion | The point of intersection of the transverse palatine suture and the median palatine suture. |
| 65 | alv | Alveolon | Most posterior point of the hard palate in the midsagittal plane. |
| 66 | ho | Hormion | The point of intersection of the vomer and sphenoid bones in the midsagittal plane. |
| 67 | ba | Basion | Most anterior point on the exterior border of the foramen magnum in the midsagittal plane. |
| 68 | o | Opisthion | Most posterior point on the exterior border of the foramen magnum in the midsagittal plane. |
| **Paired  (Right, Left)** |  |  |  |
| 6,7 | in | Infranasion | The point of intersection of the frontomaxillary and nasofrontal sutures |
| 8,9 | nm^c^ | Nasomaxillare | Most caudal point on the nasomaxillary sutures and on the nasal aperture. |
| 10,11 | al | Alare | Most lateral point on the nasal aperture in a transverse plane. |
| 12,13 | mf | Maxillofrontale | The point of intersection of the anterior lacrimal crest with the frontomaxillary suture. |
| 14,15 | mso | Mid-supraorbital | Point on the anterior aspect of the superior orbital rim, on the line that vertically bisects the orbit. |
| 16,17 | fmo | Frontomalare orbitale | The point of intersection of the orbital rim and the frontozygomatic suture. |
| 18,19 | ec | Ectoconchion | Point on the lateral orbit rim, on the line that transversely bisects the orbit. |
| 20,21 | zo | Zygoorbitale | The point of intersection of the orbital margin and the zygomaticomaxillary suture. |
| 22,23 | ifi^a^ | Infraorbital foramen inferior | Point on the lowest margin of Infraorbital foramen. |
| 24,25 | fos^a^ | Fissura orbitalis superior | The point of intersection of the superior orbital fissure and sphenofrontal suture. |
| 26,27 | oa^a^ | Orbitae apex | Point on the apex of the greater wing of the sphenoid, where the superior orbital fissure and the inferior orbital fissure meet. |
| 28,29 | foi^a^ | Fissura orbitalis inferior | The point of intersection of the inferior orbital fissure and sphenozygomatic suture. |
| 30,31 | ft | Frontotemporale | Most anteromedial point of the inferior temporal line, on the zygomatic process of the frontal bone. |
| 32,33 | fmt | Frontomalare temporale | Most lateral point on the frontozygomatic suture. |
| 34,35 | ju | Jugale | Inner vertex of the posterior zygomatic curvature, at the interaction of the vertical edge and horizontal part of the zygomatic arch |
| 36,37 | zy | Zygion | Most lateral point on the zygomatic arch. |
| 38,39 | zts^d^ | Zygotemporale superior | Most superior point of the zygomaticotemporal suture, on the upper margin of temporal process of the zygomatic bone. |
| 40,41 | zti^d^ | Zygotemporale inferior | Most inferior point of the zygomaticotemporal suture, on the lower margin of temporal process of the zygomatic bone. |
| 42,43 | zm | Zygomaxillare | Points at the lowest extent of the zygomaticomaxillary suture, not on the inferior aspect. |
| 44,45 | eci^a^ | Ectoincisor | Point on the buccal alveolar margin between the lateral incisor and canine. |
| 46,47 | ecm^e^ | Ectomolare | Lateral point on the buccal alveolar margin, at the intersection of alveolar margin and lower edge of the maxillary bone. |
| 52,53 | sphn | Sphenion | The point of intersection of the most anterior end of sphenoparietal suture and the frontal bone. |
| 54,55 | k | Krotaphion | The point of intersection of the most posterior end of sphenoparietal suture and the squamosal part of the temporal bone. |
| 56,57 | eu | Euryon | Most lateral point of the cranial vault, on the parietal bone.  (Position changes with skull shape and age) |
| 58,59 | en | Entomion | The point of intersection of the squamous and parietomastoid suture. |
| 60,61 | ast | Asterion | The point of intersection of the lambdoidal, parietomastoid, and occipitomastoid sutures. |
| 69,70 | eni^a^ | Endoincisor | Point on the lingual alveolar margin between the lateral incisor and canine. |
| 71,72 | enm^e^ | Endomolare | Most lateral point on the lingual alveolar margin, at the centre of the second molar position. |
| 73,74 | icm^d^ | Inferolateral choanal margin | The point of intersection of the lower border of the sphenoid bone and the medial plate of the pterygoid process on the posterior margin of the choanae. |
| 75,76 | scm^a^ | Superolateral choanal margin | The point of intersection of the horizontal plate of the palatine bone and the medial plate of pterygoid process on the posterior margin of the choanae. |
| 77,78 | ppa^a^ | Pterygoid process apex | Point on the inferior apex of the lateral plate of the pterygoid process. |
| 79,80 | lbo^a^ | Anterolateral basioccipital | Most anterolateral point on the basioccipital bone, close to the spheno-occiptial synchondrosis. |
| 81,82 | lfm^a^ | Lateral foramen magnum | Most lateral point on the exterior border of the foramen magnum in a plane perpendicular to the midsagittal plane. |
| 83,84 | ra | Radiculare | The point on the lateral aspect of the root of the zygomatic process at the deepest incurvature. |
| 85,86 | po | Porion | Most superior point on the upper margin of the external auditory meatus. |
| 87,88 | ms | Mastoidale | Most inferior point on the tip of the mastoid process. |
| Landmarks are positioned assuming the FH position. Common anatomical landmarks and definitions are sourced from Howells ^[S1]^ and Martin and Knussmann ^[S2]^ unless otherwise noted: a defined by authors; b after Skrzat et al. ^[S3]^; c after Evteev et al. ^[S4]^; d after Wärmländer et al. ^[S5]^; e after Caple and Stephan ^[S6]^. | | | |

**Table S2.** Summary of linear and volume measurements based on the 217 skull models from newborn to 4 years: **(A)** Linear measurements. **(B)** Key volumes. **(C)** Key indices. Note that all data are summarized by the group mean and standard deviation and are reported by sex (upper row in each cell = female, lower = male).

**A**

| **Unit: mm** | | **Sample size/Sex** | **n=4 (Female) n=6 (Male)** | **n=8 (Female) n=7 (Male)** | **n=12 (Female) n=14 (Male)** | **n=15 (Female) n=22 (Male)** | **n=14 (Female) n=19 (Male)** | **n=16 (Female) n=11 (Male)** | **n=9 (Female) n=13 (Male)** | **n=12 (Female) n=14 (Male)** | **n=4 (Female) n=17 (Male)** |  |
| --- | --- | --- | --- | --- | --- | --- | --- | --- | --- | --- | --- | --- |
| **No.** | **Region** | **Description/definition** | **0-1 Months** | **1-3 Months** | **3-6 Months** | **6-12 Months** | **12-18 Months** | **18-24 Months** | **24-30 Months** | **30-36 Months** | **36-48 Months** |  |
| **1** | **Cranial bones** | **Maximum cranial breadth (56) - (57)** | 91.0±3.5 95.9±6.3 | 103.9±6.6 105.0±4.1 | 114.6±5.3 118.9±7.4 | 120.7±6.7 124.0±5.6 | 123.1±6.2 129.2±4.9 | 127.5±4.1 133.7±4.7 | 127.5±5.6 135.8±7.5 | 128.2±4.4 136.6±6.0 | 132.2±3.1 136.5±6.7 |  |
| **2** |  | **Maximum cranial length (1) - (50)** | 110.1±5.1 112.8±5.2 | 123.0±4.7 122.3±3.4 | 129.6±4.8 130.1±6.6 | 141.0±7.3 143.0±7.0 | 146.2±9.3 152.7±7.6 | 156.9±7.3 155.5±5.6 | 156.8±5.9 158.8±7.2 | 162.1±6.1 165.0±7.2 | 162.8±5.4 166.5±6.8 |  |
| **3** |  | **Cranial circumference^a^ (1) - (50)*** | 301.4±16.7 304.6±36.1 | 334.1±33.8 331.9±23.6 | 374.7±7.3 385.3±21.1 | 408.7±19.4 419.2±21.2 | 418.9±24.2 439.8±12.9 | 452.8±24.6 455.8±20.9 | 447.6±19.3 473.7±22.2 | 457.1±17.3 472.3±18.8 | 461.8±16.5 473.3±20.5 |  |
| **4** |  | **Basion-Bregma height (67) - (48)** | 85.4±3.6 88.1±4.2 | 94.1±1.8 93.1±3.1 | 101.0±3.1 103.3±4.2 | 108.6±5.6 110.8±3.7 | 109.4±4.4 117.5±4.4 | 116.9±3.0 118.4±4.8 | 120.1±4.1 121.5±4.5 | 123.6±4.3 125.5±4.8 | 120.7±1.9 127.6±5.6 |  |
| **5** |  | **Naso-occipital length (2) - (50)** | 109.8±4.9 112.1±5.3 | 122.4±4.7 121.7±3.4 | 127.8±4.5 128.9±6.2 | 138.3±7.0 140.9±6.6 | 143.9±9.3 150.2±7.1 | 153.8±7.8 152.5±6.3 | 153.4±5.8 155.8±6.2 | 158.8±6.5 161.9±6.8 | 159.1±6.8 163.8±6.0 |  |
| **6** |  | **Cranial base length (2) - (67)** | 57.9±2.4 59.4±2.1 | 63.6±2.9 61.9±2.3 | 67.1±2.7 68.4±3.7 | 73.1±2.8 73.6±2.9 | 75.5±2.6 78.2±2.8 | 79.4±2.1 78.7±2.7 | 80.7±3.2 81.3±3.3 | 84.1±3.6 85.2±3.5 | 81.0±2.4 87.2±4.0 |  |
| **7** |  | **Prosthion-basion length^b^ (5) - (67)** | 55.4±2.1 55.5±3.6 | 58.9±2.5 58.6±1.7 | 61.2±3.4 63.1±4.1 | 66.7±2.1 67.9±3.6 | 71.0±3.3 71.3±3.1 | 74.9±3.7 73.6±2.6 | 75.4±3.2 74.5±3.0 | 77.7±4.2 77.4±3.4 | 75.8±3.2 79.4±4.1 |  |
| **8** |  | **Biasterionic breadth (60) - (61)** | 62.3±2.2 66.5±4.7 | 70.1±4.0 70.1±3.4 | 85.6±5.4 85.6±7.4 | 93.2±6.1 94.3±4.9 | 97.0±6.6 100.7±3.1 | 102.5±2.5 103.4±4.6 | 102.7±4.6 107.1±5.5 | 103.5±3.5 107.3±5.4 | 99.1±5.4 106.7±6.2 |  |
| **9** |  | **Biauricular breadth (83) - (84)** | 67.2±3.2 66.8±3.7 | 72.6±2.2 70.8±2.8 | 80.1±3.0 81.3±4.6 | 86.2±4.2 86.4±4.3 | 89.6±2.4 91.8±3.2 | 94.6±2.1 94.3±4.0 | 95.7±5.1 98.1±5.6 | 98.6±2.0 99.3±4.0 | 99.4±3.4 101.6±4.7 |  |
| **10** |  | **Mastoid height (Left) (86) - (88)** | 9.6±1.1 9.5±1.5 | 11.8±1.9 10.9±1.6 | 13.7±1.5 13.0±2.0 | 15.7±1.5 15.6±1.8 | 16.4±1.8 17.0±1.9 | 19.0±1.8 17.3±2.0 | 19.1±2.0 18.4±1.5 | 19.9±2.5 20.0±1.7 | 20.4±2.3 20.8±1.8 |  |
| **11** |  | **Frontal chord (2) - (48)** | 70.7±4.6 71.0±5.0 | 75.7±2.2 73.4±1.7 | 81.3±3.5 83.1±3.2 | 87.3±4.3 88.8±3.4 | 90.1±5.2 94.1±3.1 | 96.1±4.1 97.1±4.8 | 98.7±4.0 101.3±4.1 | 100.7±4.6 102.8±4.3 | 96.8±3.9 103.5±4.7 |  |
| **12** |  | **Parietal chord (48) - (49)** | 75.9±4.2 76.7±3.8 | 82.2±4.9 86.3±3.5 | 88.3±3.8 89.1±5.5 | 94.4±5.6 99.1±4.2 | 95.3±5.7 103.4±6.6 | 104.0±6.1 101.0±4.8 | 103.0±6.8 105.4±8.0 | 105.0±7.7 108.6±7.3 | 105.3±3.9 107.5±8.2 |  |
| **13** |  | **Occipital chord (49) - (68)** | 58.1±4.4 66.3±2.6 | 73.3±4.8 72.0±2.1 | 78.1±4.0 79.1±6.4 | 83.3±7.2 84.3±4.9 | 85.0±7.3 91.7±4.8 | 92.1±4.9 94.2±7.1 | 92.5±6.9 92.7±3.4 | 94.5±5.9 95.1±5.7 | 96.9±4.9 96.1±5.8 |  |
| **14** |  | **Foramen magnum length^c^ (Sagittal diameter) (67) - (68)** | 27.4±0.8 27.4±1.2 | 28.3±2.0 29.9±0.6 | 29.3±2.4 30.1±2.6 | 32.0±2.7 33.9±2.3 | 33.2±3.6 36.2±2.3 | 32.7±2.2 35.8±2.8 | 34.4±1.9 35.8±1.8 | 34.2±2.6 38.4±1.7 | 34.4±2.3 39.6±2.2 |  |
| **15** |  | **Foramen magnum breadth^c^ (Transverse diameter) (81) - (82)** | 19.7±1.3 19.1±1.8 | 20.0±1.5 20.8±0.8 | 22.6±1.4 23.7±2.3 | 25.0±2.1 25.9±1.9 | 26.2±1.9 27.9±2.1 | 26.8±1.9 27.8±1.4 | 26.7±1.3 28.1±1.9 | 27.9±1.8 30.0±2.0 | 26.4±1.1 30.5±1.7 |  |
| **16** | **Mid-facial region (Major facial dimensions)** | **Upper facial height (2) - (5)** | 28.2±2.7 28.9±1.6 | 31.9±1.8 30.9±1.9 | 34.6±2.0 36.1±2.3 | 39.5±2.1 39.7±2.5 | 40.8±2.5 42.4±2.1 | 44.3±2.5 44.1±2.4 | 46.0±3.6 46.2±4.0 | 48.0±3.7 49.1±2.4 | 49.0±3.5 51.9±3.0 |  |
| **17** |  | **Minimum frontal breadth (30) - (31)** | 65.8±2.5 64.2±4.7 | 69.5±3.1 68.1±2.6 | 75.4±2.9 77.1±3.7 | 77.9±5.6 79.6±3.4 | 78.1±2.9 83.3±3.3 | 82.3±1.6 84.1±4.4 | 83.4±4.5 86.7±2.6 | 85.8±2.2 87.5±2.3 | 86.5±1.5 88.4±3.4 |  |
| **18** |  | **Upper facial breadth (32) - (33)** | 63.8±2.2 65.0±4.9 | 69.0±4.1 70.2±2.3 | 75.4±2.7 76.4±4.0 | 77.4±4.6 80.2±4.0 | 77.6±2.9 82.6±3.1 | 82.0±2.0 84.2±3.7 | 81.9±4.0 86.5±2.9 | 83.7±2.6 86.9±2.4 | 86.0±2.1 88.3±3.4 |  |
| **19** |  | **Bizygomatic breadth (36) - (37)** | 70.0±2.6 69.7±4.1 | 75.7±3.2 75.2±2.5 | 82.7±3.6 83.9±5.2 | 88.8±4.6 88.8±4.6 | 90.1±3.2 94.2±3.5 | 95.4±1.7 96.9±4.1 | 95.4±4.7 99.3±4.6 | 98.9±1.8 101.7±3.0 | 101.0±2.5 103.2±4.1 |  |
| **20** | **Mid-facial region (Nasal bones)** | **Length of the nasal bones^d^ (2) - (3)** | 10.8±1.5 9.3±0.8 | 10.5±1.8 9.6±1.4 | 11.8±1.5 11.2±2.2 | 13.1±1.2 12.6±1.9 | 12.4±1.6 13.7±1.4 | 14.0±1.2 13.8±2.1 | 14.6±1.9 14.3±1.7 | 15.8±2.0 15.5±2.4 | 14.6±1.4 15.6±2.9 |  |
| **21** |  | **Upper breadth of the nasal bones^d^ (6) - (7)** | 8.5±1.1 9.4±1.2 | 10.1±1.8 9.5±0.7 | 11.4±1.5 10.9±1.1 | 11.4±1.3 11.0±1.6 | 11.5±1.3 11.3±1.5 | 11.0±0.8 10.9±1.7 | 12.6±0.8 11.5±1.7 | 12.5±1.1 11.8±1.3 | 11.0±1.0 11.9±1.7 |  |
| **22** |  | **Lower breadth of the nasal bones^d^ (8) - (9)** | 9.9±0.7 11.1±1.2 | 11.6±1.2 11.9±0.4 | 12.1±0.9 12.4±1.1 | 12.8±1.4 13.1±1.4 | 12.9±0.8 12.9±1.6 | 12.8±1.5 13.9±1.2 | 14.0±1.8 13.7±1.1 | 13.5±1.5 13.3±1.0 | 12.1±2.2 14.1±1.5 |  |
| **23** |  | **Length of nasomaxillary suture (left)^d^ (7) - (9)** | 12.1±1.8 11.3±0.8 | 12.1±1.6 12.4±1.1 | 12.7±1.2 13.7±1.0 | 14.8±1.2 14.8±1.8 | 14.7±1.5 15.9±1.3 | 15.5±1.2 16.6±2.0 | 17.0±2.4 16.6±1.4 | 17.3±2.1 17.5±1.5 | 16.4±0.7 18.7±2.2 |  |
| **24** |  | **Nasal breadth^d^ (10) - (11)** | 14.9±0.2 14.6±0.7 | 15.9±1.2 16.1±1.2 | 17.1±0.9 17.1±1.5 | 18.0±1.3 18.4±1.4 | 18.3±1.3 19.0±1.6 | 19.2±1.0 19.7±1.5 | 20.0±1.6 20.0±1.1 | 19.6±1.5 20.4±1.2 | 19.5±1.5 20.6±1.7 |  |
| **25** |  | **Nasal height^d^ (2) - (4)** | 22.3±2.5 22.2±1.2 | 24.5±1.5 23.4±1.5 | 26.3±1.8 26.8±1.7 | 29.1±1.4 29.3±1.7 | 30.6±1.5 31.8±1.7 | 32.3±1.9 32.3±1.6 | 32.8±3.0 33.7±2.6 | 35.3±3.5 36.0±2.0 | 34.2±2.3 37.3±2.3 |  |
| **26** |  | **Nasal cavity length^e^ (4) - (65)** | 27.7±0.7 26.5±1.1 | 30.0±2.6 27.9±2.2 | 30.0±2.4 30.9±2.1 | 32.5±1.2 31.6±1.8 | 33.6±1.8 32.8±2.7 | 35.3±2.1 33.0±1.6 | 36.7±1.9 34.6±1.7 | 37.3±2.7 36.7±2.1 | 37.4±2.4 37.5±2.4 |  |
| **27** |  | **Nasal cavity height^e^ (65) - (62)** | 19.8±0.9 19.9±2.6 | 22.2±2.6 24.5±1.7 | 26.1±1.8 28.3±2.1 | 28.9±1.7 30.6±1.8 | 31.0±2.6 31.9±1.8 | 33.0±1.9 33.6±1.5 | 32.3±3.3 35.2±3.0 | 35.1±2.3 37.0±2.7 | 36.0±0.6 36.8±2.6 |  |
| **28** |  | **Upper breadth of choana^e^ (75) - (76)** | 8.7±0.8 7.1±0.8 | 9.3±1.5 8.4±1.1 | 9.3±0.8 8.0±0.8 | 9.6±1.4 9.3±1.2 | 10.1±1.6 10.4±1.7 | 10.4±1.2 11.5±1.4 | 10.8±0.9 11.1±1.5 | 10.1±0.5 12.1±1.1 | 11.8±1.7 12.7±1.4 |  |
| **29** |  | **Lower breadth of choana^e^ (73) - (74)** | 13.8±1.0 13.5±0.6 | 14.5±1.8 14.6±1.3 | 15.4±1.8 15.0±1.5 | 16.7±1.5 16.3±1.3 | 16.7±1.3 17.0±1.2 | 17.3±0.9 17.7±1.4 | 18.1±0.9 18.4±1.4 | 17.2±1.7 18.6±1.4 | 19.4±0.5 19.8±1.5 |  |
| **30** |  | **Height of the choana^d^ (65) - (66)** | 8.8±1.0 10.2±1.5 | 10.3±1.2 11.6±0.9 | 10.9±0.9 12.9±1.2 | 13.7±1.7 15.1±1.3 | 15.2±1.2 16.0±2.3 | 16.1±1.8 16.9±1.3 | 16.1±1.8 17.6±2.3 | 17.0±1.6 19.4±1.7 | 17.7±1.2 19.9±1.7 |  |
| **31** | **Mid-facial region (Orbital bones)** | **Orbital breadth (Left)^d^ (13) - (19)** | 24.9±1.2 25.1±2.2 | 27.1±0.9 28.2±1.6 | 29.7±1.7 29.9±2.1 | 30.9±2.1 31.5±1.4 | 31.1±1.0 33.0±1.8 | 32.5±1.2 33.9±1.5 | 32.2±1.9 34.9±1.9 | 32.5±1.0 34.8±1.2 | 34.7±1.3 35.7±1.3 |  |
| **32** |  | **Orbital breadth (Right)^d^ (12) - (18)** | 25.1±1.4 25.0±1.7 | 27.1±1.5 27.8±1.3 | 30.0±1.6 29.8±2.1 | 31.3±1.8 31.6±1.4 | 31.6±0.6 33.5±1.7 | 32.6±1.0 33.7±1.7 | 32.3±1.4 34.4±2.0 | 32.3±1.4 35.3±1.4 | 34.6±1.0 35.7±1.3 |  |
| **33** |  | **Orbital height (Left)^d^ (15) - (21)** | 21.0±2.1 21.3±2.3 | 22.9±1.0 23.6±1.0 | 26.0±0.9 26.7±2.1 | 28.2±2.1 28.1±1.7 | 28.8±1.4 29.8±2.4 | 28.8±1.8 30.1±2.0 | 30.0±2.3 30.4±2.2 | 30.3±2.0 31.6±2.0 | 29.3±1.8 32.2±1.8 |  |
| **34** |  | **Orbital height (Right)^d^ (14) - (20)** | 20.7±1.9 20.7±2.2 | 22.8±0.8 23.6±1.3 | 25.5±0.9 26.5±1.9 | 28.2±2.2 27.9±1.7 | 28.3±1.5 29.6±2.0 | 28.6±1.9 30.0±1.9 | 29.2±2.4 30.2±1.9 | 30.0±2.1 31.4±2.1 | 29.0±1.6 32.1±1.7 |  |
| **35** |  | **Interorbital breadth  (12) - (13)** | 15.1±1.3 16.5±1.2 | 17.0±1.7 17.2±0.7 | 18.0±1.2 19.0±1.7 | 18.2±2.1 18.6±1.8 | 17.4±1.4 18.7±1.5 | 18.5±1.3 18.8±2.1 | 19.2±1.8 19.0±1.5 | 19.8±1.6 19.6±1.2 | 17.8±0.6 19.4±1.9 |  |
| **36** |  | **Biorbital breadth (18) - (19)** | 61.7±2.6 61.3±4.1 | 66.6±3.7 67.1±3.1 | 72.1±3.3 71.8±4.9 | 73.9±3.7 74.2±3.8 | 72.8±2.5 75.8±3.2 | 76.6±1.6 77.3±3.4 | 74.8±4.2 78.1±3.1 | 77.0±2.0 79.1±2.7 | 79.3±0.8 80.0±3.1 |  |
| **37** | **Mid-facial region (Maxilla bones)** | **Zygoorbitale chord^d^ (20) - (21)** | 30.7±1.8 34.6±2.8 | 35.9±3.3 38.1±3.8 | 38.9±2.8 41.5±3.3 | 39.2±2.4 43.2±2.3 | 39.1±3.2 43.0±3.5 | 40.2±2.0 43.0±3.4 | 41.8±3.6 43.5±3.0 | 42.0±2.9 43.1±2.3 | 42.0±2.5 42.5±3.8 |  |
| **38** |  | **Bimaxillary breadth (42) - (43)** | 54.0±1.9 52.5±3.8 | 58.6±2.3 57.5±2.5 | 61.3±3.4 62.1±3.7 | 65.8±3.9 66.2±4.1 | 65.4±3.8 68.6±3.4 | 69.7±2.0 70.3±4.3 | 70.8±3.4 72.8±4.4 | 74.9±3.0 73.5±3.3 | 72.7±2.3 76.8±3.3 |  |
| **39** |  | **Maximum Maxillo-alveolar breadth (46) - (47)** | 39.1±1.1 38.9±2.2 | 41.4±1.7 41.9±1.0 | 42.0±1.5 43.0±2.8 | 43.6±2.3 45.2±2.5 | 43.9±2.5 46.9±2.8 | 47.4±1.7 48.4±2.9 | 48.0±3.1 50.4±2.4 | 48.7±2.9 50.5±2.1 | 49.9±1.3 51.8±2.4 |  |
| **40** |  | **Maxillo-alveolar breadth 2^e^ (44) - (45)** | 23.0±1.6 22.2±1.2 | 23.2±1.6 25.0±1.6 | 24.2±1.8 26.0±1.5 | 25.2±1.9 26.2±1.9 | 25.1±1.4 24.7±1.4 | 24.5±1.5 25.1±1.3 | 24.3±2.3 25.1±1.2 | 24.3±1.7 24.9±1.1 | 23.8±0.5 24.9±1.1 |  |
| **41** |  | **Maximum alveolar length (5) - (65)** | 27.9±0.6 27.5±1.4 | 29.3±2.6 28.7±2.3 | 30.5±3.1 31.5±2.2 | 33.4±1.5 33.8±2.4 | 35.2±2.5 35.1±2.4 | 37.9±2.1 36.9±1.8 | 39.2±1.8 37.6±1.9 | 39.8±3.2 39.2±2.4 | 38.6±1.0 40.4±2.3 |  |
| **42** |  | **Anterior height of the alveolar process^d^ (4) - (5)** | 6.0±1.3 6.7±0.6 | 7.7±1.4 7.6±0.8 | 8.4±0.9 9.3±1.0 | 10.5±1.7 10.7±1.4 | 10.4±2.3 10.9±2.0 | 12.2±2.2 12.4±1.2 | 13.4±2.0 12.8±2.1 | 12.9±1.8 13.5±2.1 | 15.1±1.1 14.8±1.7 |  |
| **43** | **Mid-facial region (Zygomatic)** | **Zygomatic height (Left)^f^ (33) - (43)** | 19.0±2.1 19.6±2.0 | 21.8±1.5 21.5±1.8 | 24.9±2.0 26.3±1.6 | 27.9±1.8 27.6±1.5 | 29.5±2.1 29.7±1.6 | 31.2±2.2 31.7±2.5 | 30.5±3.2 32.3±1.8 | 32.5±2.4 35.0±1.5 | 33.6±1.1 35.7±2.9 |  |
| **44** |  | **Zygomatic chord (Left)^f^ (21) - (41)** | 29.0±1.9 27.6±1.9 | 30.7±2.7 31.0±1.9 | 33.4±2.0 32.5±2.1 | 36.2±2.9 35.8±2.6 | 38.2±1.8 38.8±2.3 | 40.3±2.1 39.9±1.8 | 39.6±2.1 41.7±2.9 | 41.0±2.3 43.5±0.9 | 43.9±2.1 45.2±2.6 |  |
| **45** | **Mid-facial region (major palatal dimensions)** | **Maximum palatal length^e^ (63) - (65)** | 23.5±2.0 21.1±2.3 | 23.2±2.7 22.7±2.5 | 24.8±2.9 25.5±2.2 | 28.4±2.5 28.7±3.2 | 31.3±2.9 31.5±2.9 | 34.4±2.4 34.0±1.6 | 35.4±2.0 34.6±1.8 | 36.3±2.5 36.0±2.9 | 35.8±1.2 36.9±2.6 |  |
| **46** |  | **Internal Palatal width 1^e^ (Inter flateral incisor) (69) - (70)** | 15.3±0.6 16.0±1.3 | 16.6±1.8 16.7±2.1 | 16.8±1.6 17.1±2.0 | 17.9±1.7 18.9±2.0 | 18.1±1.6 18.4±2.0 | 18.2±1.6 18.6±2.0 | 17.6±2.1 18.9±1.4 | 17.5±1.6 18.4±1.2 | 18.4±2.7 18.0±1.4 |  |
| **47** |  | **Internal Palatal width 2^e^ (Inter second molar) (71) - (72)** | 21.5±2.3 19.3±1.4 | 22.8±1.7 21.2±2.3 | 22.7±2.4 22.2±2.0 | 24.1±2.0 23.7±2.3 | 23.8±2.1 23.6±1.6 | 24.2±1.8 24.5±2.1 | 24.1±2.4 25.1±2.0 | 23.9±2.0 25.0±1.2 | 26.1±0.9 25.6±2.0 |  |
| Cranial measurements are sourced from Howells ^[S1]^ and Martin and Knussmann ^[S2]^ unless otherwise noted: a: after Libby et al. ^[S7]^; b: after Lesciotto et al. ^[S8]^; c: after Gruber et al. ^[S9]^; d: after Evteev et al. ^[S4]^; e: defined by authors; f: after Nikolova et al. ^[S10]^.  * that which passes through the landmark point 1 and 50 and is perpendicular to the midsagittal plane. | | | | | | | | | | | | |

**B**

| **Unit: cm^3^** | | **Sample size/Sex** | **n=4 (Female) n=6 (Male)** | **n=8 (Female) n=7 (Male)** | **n=12 (Female) n=14 (Male)** | **n=15 (Female) n=22 (Male)** | **n=14 (Female) n=19 (Male)** | **n=16 (Female) n=11 (Male)** | **n=9 (Female) n=13 (Male)** | **n=12 (Female) n=14 (Male)** | **n=4 (Female) n=17 (Male)** |
| --- | --- | --- | --- | --- | --- | --- | --- | --- | --- | --- | --- |
| **No.** | **Definition** | **Description** | **0-1 Months** | **1-3 Months** | **3-6 Months** | **6-12 Months** | **12-18 Months** | **18-24 Months** | **24-30 Months** | **30-36 Months** | **36-48 Months** |
| **1** | **Key Volumes** | **Intracranial volume (ICV)^a^** | 448.1±60.9 486.2±77.9 | 624.3±47.4 629.0±37.1 | 768.4±75.7 809.4±90.7 | 936.7±124.9 1001.6±102.0 | 956.8±132.7 1140.0±72.9 | 1135.3±87.2 1177.3±96.4 | 1179.9±91.5 1263.9±127.3 | 1211.8±135.2 1341.0±113.5 | 1192.9±34.3 1349.4±117.3 |
| **2** |  | **Left orbital volume (LOV)^b^** | 6.5±1.4 6.4±1.2 | 8.5±0.9 8.8±0.7 | 11.1±1.1 11.1±1.7 | 13.8±1.8 14.0±1.3 | 14.4±1.2 15.7±1.1 | 16.1±1.4 17.0±1.4 | 15.8±2.0 17.3±1.9 | 16.7±1.7 18.9±1.5 | 17.0±1.1 19.5±1.4 |
| **3** |  | **Nasal cavity volume (NCV)^b^** | 4.1±0.4 3.9±0.5 | 5.6±0.4 5.0±0.2 | 6.2±0.6 6.6±1.0 | 7.7±1.1 8.5±1.1 | 8.4±1.0 9.9±1.4 | 10.4±1.2 11.3±1.5 | 11.9±2.0 12.3±1.4 | 13.3±1.8 14.2±1.0 | 14.1±1.4 15.8±2.2 |
| **4** |  | **Upper intraoral volume (UIV)^b^** | 0.9±0.2 0.9±0.3 | 1.3±0.2 1.3±0.1 | 1.5±0.3 1.5±0.4 | 2.0±0.4 2.1±0.4 | 2.3±0.5 2.4±0.5 | 2.9±0.5 2.6±0.4 | 3.3±0.6 2.9±0.4 | 3.4±0.8 3.2±0.5 | 3.7±0.8 3.9±0.8 |
| a: after Libby et al. ^[S7]^; b: defined by authors. | | | | | | | | | | |  |

**C**

|  | | **Sample size/Sex** | **n=4 (Female) n=6 (Male)** | **n=8 (Female) n=7 (Male)** | **n=12 (Female) n=14 (Male)** | **n=15 (Female) n=22 (Male)** | **n=14 (Female) n=19 (Male)** | **n=16 (Female) n=11 (Male)** | **n=9 (Female) n=13 (Male)** | **n=12 (Female) n=14 (Male)** | **n=4 (Female) n=17 (Male)** |
| --- | --- | --- | --- | --- | --- | --- | --- | --- | --- | --- | --- |
| **No.** | **Definition** | **Description** | **0-1 Months** | **1-3 Months** | **3-6 Months** | **6-12 Months** | **12-18 Months** | **18-24 Months** | **24-30 Months** | **30-36 Months** | **36-48 Months** |
| **1** | **Key indices** | **Cranial Module (CM)** | 95.5±3.9 98.9±4.8 | 107.0±3.2 106.8±1.6 | 115.0±2.8 117.4±4.9 | 123.4±5.4 125.9±3.9 | 126.3±5.8 133.1±3.0 | 133.8±2.7 135.8±3.4 | 134.8±4.3 138.7±4.3 | 138.0±4.0 142.4±4.0 | 138.6±1.9 143.5±3.7 |
| **2** |  | **Cephalic Index (CI)** | 82.7±1.7 85.0±4.3 | 84.5±4.8 86.0±5.0 | 88.6±5.6 91.5±6.1 | 85.8±5.0 86.9±5.9 | 84.4±4.3 84.8±6.3 | 81.5±5.2 86.1±4.9 | 81.4±3.6 85.7±6.6 | 79.2±3.0 83.0±5.7 | 81.3±4.0 82.2±5.9 |
| **3** |  | **Cranio-Facial Index (CFI)** | 77.0±0.8 72.8±3.4 | 73.1±5.7 71.7±3.6 | 72.2±2.2 70.6±2.9 | 73.6±2.4 71.6±2.6 | 73.3±2.5 73.0±2.6 | 74.9±2.4 72.5±1.6 | 74.8±2.8 73.2±2.9 | 77.2±3.2 74.5±2.6 | 76.5±3.3 75.7±2.4 |
| **4** |  | **Upper Facial Index (UFI)** | 40.1±2.3 41.4±1.2 | 42.2±2.4 41.1±2.5 | 41.8±1.5 43.1±2.2 | 44.5±2.3 44.8±2.8 | 45.3±2.7 45.1±2.5 | 46.5±2.5 45.6±3.1 | 48.3±3.5 46.5±3.0 | 48.6±3.5 48.4±2.9 | 48.5±3.0 50.3±2.5 |
| **5** |  | **Left Orbital Index (LOI)** | 84.2±5.4 84.8±4.5 | 84.7±3.8 83.8±4.7 | 87.8±5.8 89.8±9.7 | 91.3±4.4 89.4±5.3 | 92.8±4.9 90.3±6.5 | 88.7±4.7 88.6±3.9 | 93.0±5.2 87.2±4.7 | 93.0±4.5 90.9±6.3 | 84.4±4.1 90.4±5.5 |
| **6** |  | **Nasal Index (NI)** | 67.4±6.4 66.0±3.6 | 65.1±5.3 69.2±8.1 | 65.4±5.1 63.9±5.4 | 61.7±4.4 63.0±4.8 | 59.8±3.9 59.8±4.9 | 59.5±4.1 61.5±6.9 | 61.5±5.9 59.8±5.7 | 55.8±6.5 56.8±4.6 | 57.2±5.2 55.6±5.8 |
| **7** |  | **Palatal Index (PI)** | 92.8±16.3 92.1±9.2 | 100.4±18.9 95.1±17.2 | 92.5±13.3 87.7±9.8 | 85.6±10.0 83.9±13.2 | 76.7±8.2 75.6±8.1 | 70.6±6.1 72.2±7.1 | 68.1±6.2 72.5±5.0 | 66.1±7.9 69.7±6.2 | 72.9±3.3 69.8±5.9 |
| **8** |  | **Foramen Magnum Index (FMI)** | 71.8±2.6 69.7±5.8 | 71.0±9.0 69.7±3.6 | 77.8±8.6 79.1±7.9 | 78.3±6.6 76.7±6.4 | 79.9±9.1 77.5±7.9 | 82.1±4.6 78.0±6.8 | 77.9±4.1 78.5±5.0 | 81.7±4.7 78.2±4.4 | 76.7±3.0 77.1±5.0 |
| *The detailed definitions of each index are shown below: Cranial Module = (Maximum cranial length + Maximum cranial breadth + Basion-Bregma height) / 3; Cephalic Index = Maximum cranial breadth / Maximum cranial length x 100; Cranio-Facial Index = Bizygomatic breadth / Maximum cranial breadth x 100; Upper Facial Index = Upper facial height / Bizygomatic breadth x 100; Left Orbital Index = Orbital height (Left) / Orbital breadth (Left) x 100; Nasal Index = Nasal breadth / Nasal height x 100; Palatal Index = Internal Palatal width 2 / Maximum palatal length x 100; Foramen Magnum Index = Foramen magnum breadth / Foramen magnum length x 100; | | | | | | | | | | | |

**Table S3**. Comparisons of scaling of cranial volumes between sexes. Angles are calculated between linear regression vectors of cube roots of each cranial volume (ICV: intracranial volume; NCV: nasal cavity volume; LOV: left orbital volume; UIV: upper intraoral volume) on the natural logarithm of centroid size (Ln(CS)) and age in months. p-values estimated from permutation tests (n=1000).

| **Linear regression** | **Females** | | **Males** | | **Ontogenetic vector comparisons** | |
| --- | --- | --- | --- | --- | --- | --- |
|  | **R^2^** | **p-value** | **R^2^** | **p-value** | **Angle (°)** | **p-value** |
| ICV vs. Ln(CS) | 0.98 | 0.001 *** | 0.98 | 0.001 *** | 0.16 | 0.212 |
| NCV vs. Ln(CS) | 0.78 | 0.001 *** | 0.85 | 0.001 *** | 3.37 | 0.022 * |
| LOV vs. Ln(CS) | 0.87 | 0.001 *** | 0.91 | 0.001 *** | 2.45 | 0.014 * |
| UIV vs. Ln(CS) | 0.72 | 0.001 *** | 0.74 | 0.001 *** | 0.48 | 0.833 |
| ICV vs. age | 0.66 | 0.001 *** | 0.66 | 0.001 *** | <0.01 | 0.577 |
| NCV vs. age | 0.84 | 0.001 *** | 0.82 | 0.001 *** | <0.01 | 0.94 |
| LOV vs. age | 0.58 | 0.001 *** | 0.66 | 0.001 *** | <0.01 | 0.659 |
| UIV vs. age | 0.67 | 0.001 *** | 0.72 | 0.001 *** | <0.01 | 0.288 |
| Significance: ‘***’ p≤0.001, ‘**’ p≤0.01, ‘*’ p≤0.05. | | | | | | |

**Table S4.** Partial Least Squares analysis of covariations between cranial form (Block1) and cranial volumes after cube root transformation (CVols-Block2), and between cranial shape, facial shape and neurocranial shape (Block1) and cranial volume proportions after cube root transformation (CPVols-Block2). Correlations of scores on the first PLS axes and their significance are given together with the percentages of variance of Block1 explained by its association with Block2. Note that each line of the table corresponds to a separate PLS.

| **Cranial form** | **Corr. Coefficient, r** | **p-value** | **% Var. Block 1** |
| --- | --- | --- | --- |
| All cranial volumes (CVols- ICV, LOV, NCV and UIV) | 0.99 | 0.001 *** | 84.8 |
| Intracranial volume (CICV) | 0.99 | 0.001 *** | 84.8 |
| Left orbital volume (CLOV) | 0.95 | 0.001 *** | 84.8 |
| Nasal cavity volume (CNCV) | 0.91 | 0.001 *** | 84.7 |
| Upper intraoral volume (CUIV) | 0.85 | 0.001 *** | 84.7 |
| **Cranial shape** | **Corr. Coefficient, r** | **p-value** | **% Var. Block 1** |
| Proportions of ICV, LOV, NCV and UIV (CPVols) | 0.66 | 0.001 *** | 21.0 |
| Proportion of ICV (CPICV) | 0.65 | 0.001 *** | 20.5 |
| Proportion of LOV (CPLOV) | 0.41 | 0.014 * | 6.8 |
| Proportion of NCV (CPNCV) | 0.58 | 0.001 *** | 19.9 |
| Proportion of UIV (CPUIV) | 0.50 | 0.001 *** | 23.5 |
| **Facial shape** | **Corr. Coefficient, r** | **p-value** | **% Var. Block 1** |
| Proportions of ICV, LOV, NCV and UIV (CPVols) | 0.61 | 0.001 *** | 32.0 |
| Proportion of ICV (CPICV) | 0.57 | 0.001 *** | 31.4 |
| Proportion of LOV (CPLOV) | 0.31 | 0.011 * | 15.7 |
| Proportion of NCV (CPNCV) | 0.54 | 0.001 *** | 31.5 |
| Proportion of UIV (CPUIV) | 0.53 | 0.001 *** | 31.5 |
| **Neurocranial shape** | **Corr. Coefficient, r** | **p-value** | **% Var. Block 1** |
| Proportions of ICV, LOV, NCV and UIV (CPVols) | 0.60 | 0.001 *** | 18.4 |
| Proportion of ICV (CPICV) | 0.58 | 0.001 *** | 18.1 |
| Proportion of LOV (CPLOV) | 0.34 | 0.071 | 7.3 |
| Proportion of NCV (CPNCV) | 0.55 | 0.001 *** | 17.0 |
| Proportion of UIV (CPUIV) | 0.47 | 0.001 *** | 20.4 |
| Significance: ‘***’ p≤0.001, ‘**’ p≤0.01, ‘*’ p≤0.05 | | | |

**Table S5.** Results from Partial Least Squares analysis of covariations between cranial form (Block1) and cranial volumes (CVols-Block2), between cranial shape, facial shape and neurocranial shape (Block1) and volume proportions after cube root transformation (CPVols-Block2), and the correlations between all three PLS axes for Block2 and Ln(CS). Note: where signs of the correlations are negative this is because the directions of the PLS axes are arbitrary.

|  | **Cranial form (Block1) vs. CVols (Block2)** | | | **Correlation with Ln(CS)** | |
| --- | --- | --- | --- | --- | --- |
|  | **Corr. Coefficient, r** | **p-value** | **% Covariance** | **Corr. Coefficient, r** | **p-value** |
| PLS axis 1 | 0.99 | 0.001 *** | >99.9 | -0.98 | < 0.001 *** |
| PLS axis 2 | 0.65 | 0.059 | <0.1 | 0.01 | 0.942 |
| PLS axis 3 | 0.31 | 0.278 | <0.1 | -0.01 | 0.844 |
|  | **Cranial shape (Block1) vs. CPVols (Block2)** | | | **Correlation with Ln(CS)** | |
|  | **Corr. Coefficient, r** | **p-value** | **% Covariance** | **Corr. Coefficient, r** | **p-value** |
| PLS axis 1 | 0.66 | 0.001 *** | 96.7 | -0.63 | 0.001 *** |
| PLS axis 2 | 0.39 | 0.010 * | 2.3 | -0.47 | 0.001 *** |
| PLS axis 3 | 0.22 | 0.001 *** | 1.0 | <0.01 | 0.957 |
|  | **Facial shape (Block1) vs. CPVols (Block2)** | | | **Correlation with Ln(CS)** | |
|  | **Corr. Coefficient, r** | **p-value** | **% Covariance** | **Corr. Coefficient, r** | **p-value** |
| PLS axis 1 | 0.61 | 0.001 *** | 97.2 | -0.76 | < 0.001 *** |
| PLS axis 2 | 0.50 | 0.011 * | 1.9 | -0.42 | < 0.001 *** |
| PLS axis 3 | 0.44 | 0.001 *** | 0.9 | 0.20 | 0.003 ** |
|  | **Neurocranial shape (Block1) vs. CPVols (Block2)** | | | **Correlation with Ln(CS)** | |
|  | **Corr. Coefficient, r** | **p-value** | **% Covariance** | **Corr. Coefficient, r** | **p-value** |
| PLS axis 1 | 0.60 | 0.001 *** | 96.2 | 0.66 | < 0.001 *** |
| PLS axis 2 | 0.35 | 0.075 | 2.2 | -0.23 | < 0.001 *** |
| PLS axis 3 | 0.24 | 0.001 *** | 1.6 | -0.01 | 0.903 |
| Significance: ‘***’ p≤0.001, ‘**’ p≤0.01, ‘*’ p≤0.05. | | | | | |

**Figure S1. (A)-(C)** Surface regions for placing the surface SLMs: **(A)** Anterior view of the midfacial region. **(B)** inferior view showing the lower boundaries of cranial semilandmarks. **(C)** The selected regions of exoccipital and basioccipital bones without occipital condyle. Note that all the gaps between bones (e.g. sutures), fossae, protuberances or holes (i.e. infraorbital foramen – see white arrow on Supplementary Fig. S1A) have been manually filled or modified; **(D)-(G)** Boundaries for the segmentation of four cranial volumes: **(D)** Intracranial volume. **(E)** Bony orbital volume. **(F)** Bony nasal cavity volume. **(G)** Upper intraoral volume. Note the landmarks used to define these boundaries 1: Upper inferior limit of intracranial volume at the cribriform plate; 2: Lower inferior limit of intracranial volume at Foramen magnum; 3: Lower anterior boundary of intracranial volume at posterior edge of sphenoid bone; 4: Lateral orbital rim; 5: Medial orbital rim; 6: Medial orbital wall; 7: The most anterior portion of optic canal; 8: Upper limit of maxillary sinus; 9: Rhinion; 10: Nasospinale; 11: The most posterior limit of palatine bone; 12: Hormion; 13: Upper limit of the nasal cavity (below Nasion point); 14: Nasal floor; 15: lateral nasal wall; 16: Orale; 17: The most posterior point on the lingual aspect of the alveolar process.


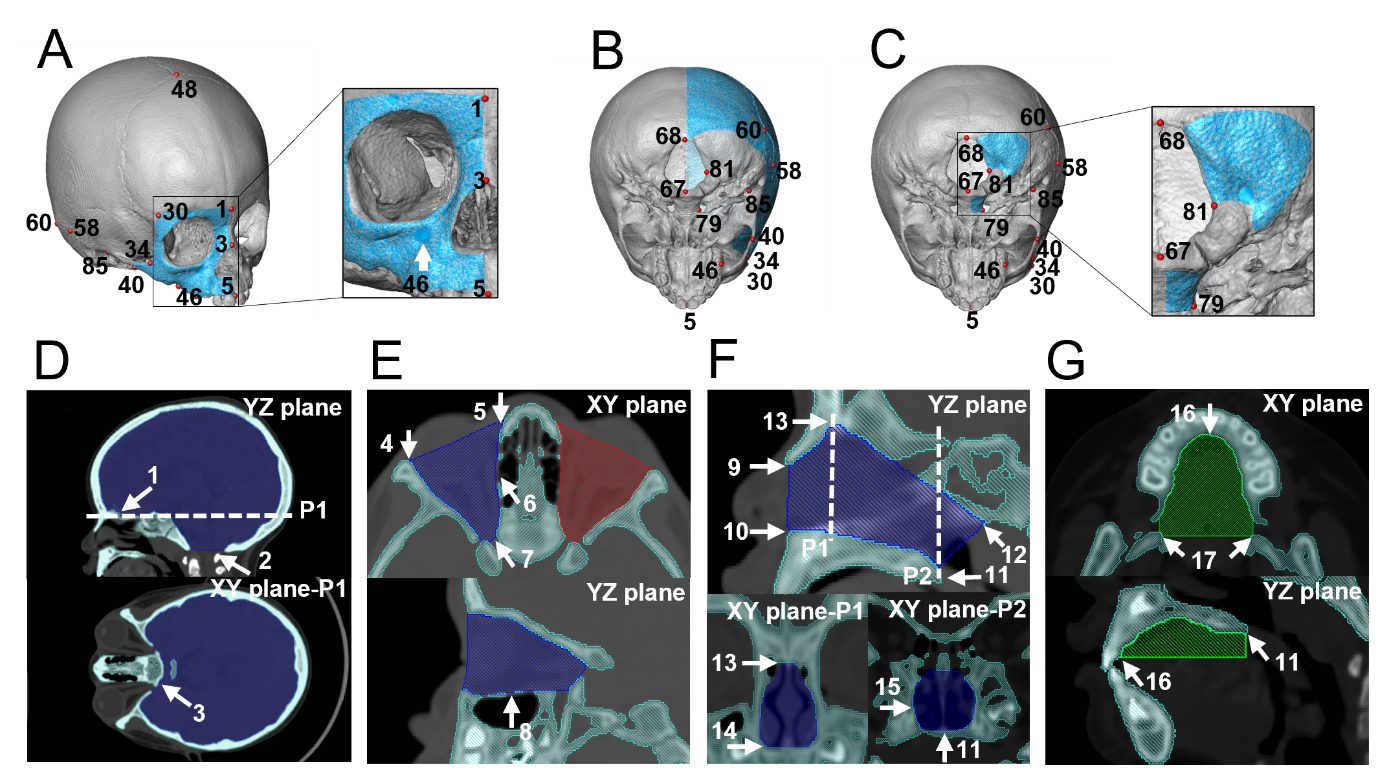


**Figure S2.** Determination of the ranges of Ln(CS) corresponding to 9 age groups: **(A)** Boxplot of the Ln(CS) in the 9 age groups. p-values are assessed via t-test between sexes in each age group. Significance: ‘***’ p≤0.001, ‘**’ p≤0.01, ‘*’ p≤0.05, ‘ns’ p>0.05. **(B)** Form space PCA analysis (same analysis as presented in main text Fig.4) of all 217 individuals used to compute means of 9 age groups (in months) with combined sexes and corresponding 9 ranges of Ln(CS). **(C)** Sum of the Procrustes distances between the mean cranial surfaces of females (green outline) and males (red outline) for successive age groups, and between the surfaces corresponding to the mean Ln(CS) of successive age groups. Procrustes distances are calculated between corresponding nodes of mean male and female cranial surfaces.


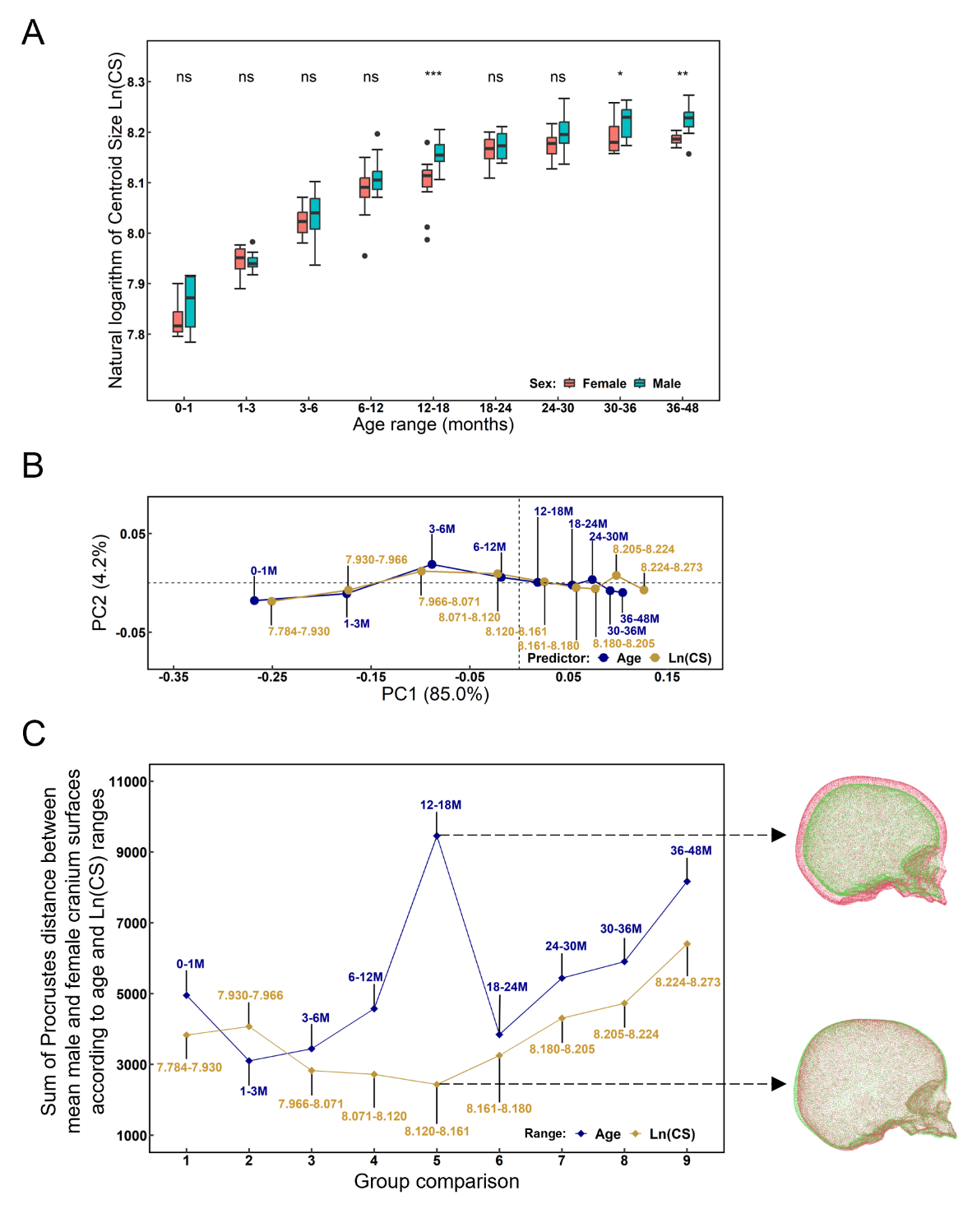


**Figure S3. (A)** Two-block partial least squares (PLS) analysis between cranial form (Ln(CS) plus shape variables - Block1) and cranial volumes (ICV, LOV, NCV and UIV) after cube root transformation (CVols - Block2). See the caption to Figure 6 for details of template surfaces reported below. **(B)** Loadings of the of each cranial volume after cube root transformation (CICV, CLOV, CNCV and CUIV – Block2) on the first PLS axis. **(C)** The same PLS analysis between cranial form (Block1) and CVols (Block2), with the means of each of the 9 age groups (in months) plotted by sex plotted. **(D)** PLS between cranial shape (Block1) and CPVols (Block2). **(E)** PLS between facial shape (Block1) and CPVols (Block2). **(F)** PLS between neurocranial shape (Block1) and CPVols (Block2). Note that (D)-(F) are related to Figure 6 (A)-(C) respectively.


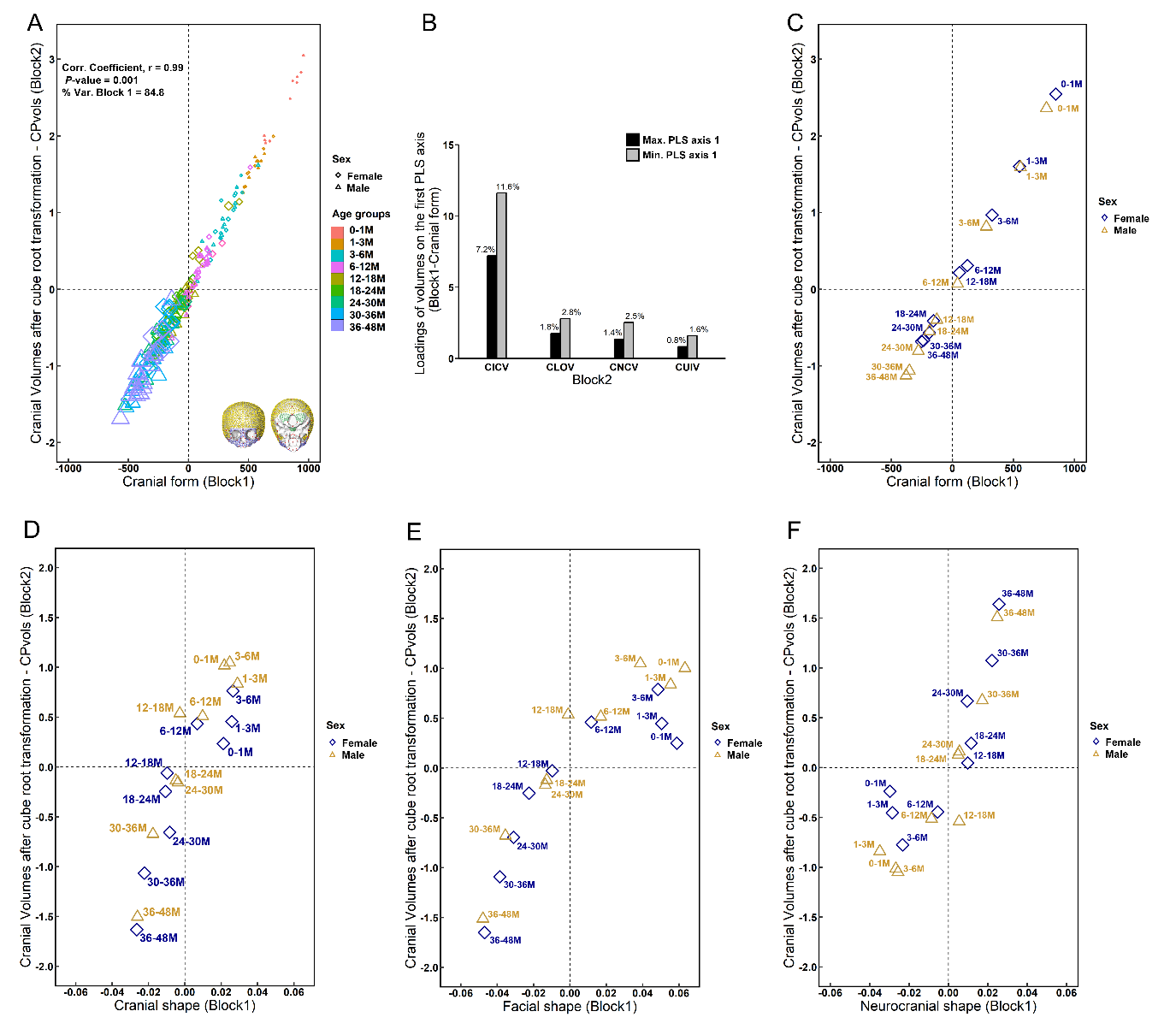


**Figure S4.** Colour maps of surface area changes between the cranial surfaces derived by: **(A)** regression of shape on the minimum Ln(CS) and the maximum score on PLS axis 1 which relate to the smallest/youngest specimens (The range of surface area difference is from -29.4% to 25.5%). **(B)** regression of shape on the maximum Ln(CS) and the minimum PLS axis 1 which relate to the largest/oldest specimens (The range of surface area difference is from -25.2% to 38.1%). Note that the color map (third column) indicates the relative surface area changes between the target surface (second column) with respect to reference surface (first column). Positive percentage value represents the relative expansion of specific regions from the reference surface (first column) to the target surface (second column). The two black lines displayed on each right-side scalebar show the limits of surface area differences in expansion or contraction.


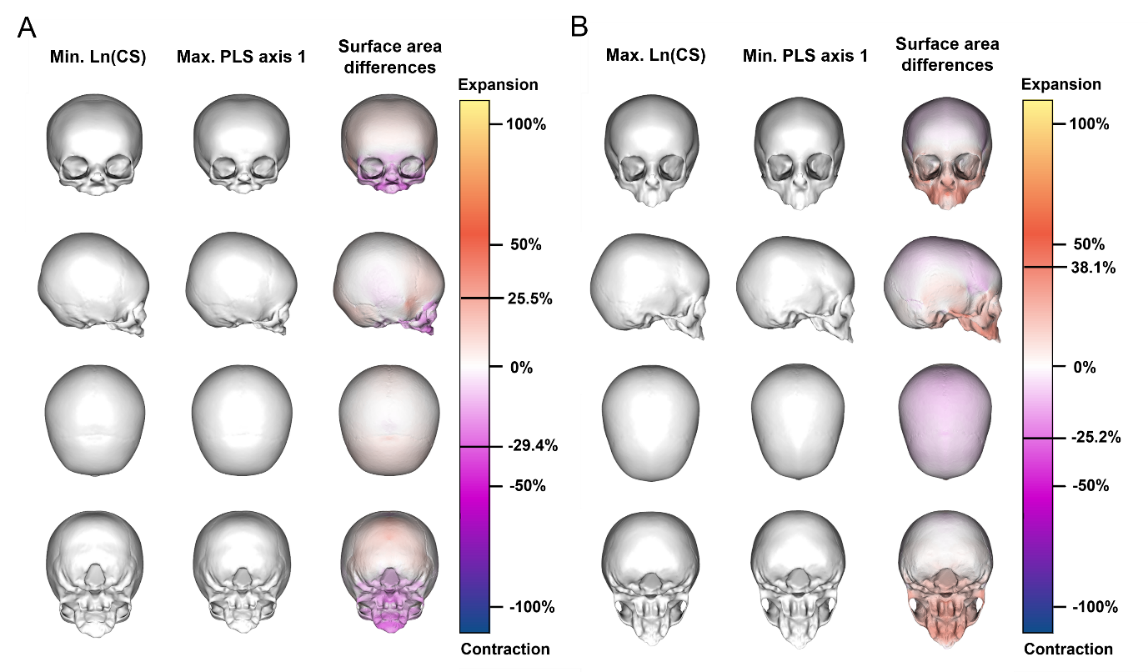


**Figure S5.** Absolute differences between the mean left and right orbital volumes (LOV and ROV) based on 21 males and females randomly selected from 217 individuals throughout the entire age range (0 to 4 years). Note that all these orbital volumes were manually segmented based on the CT scans.

**
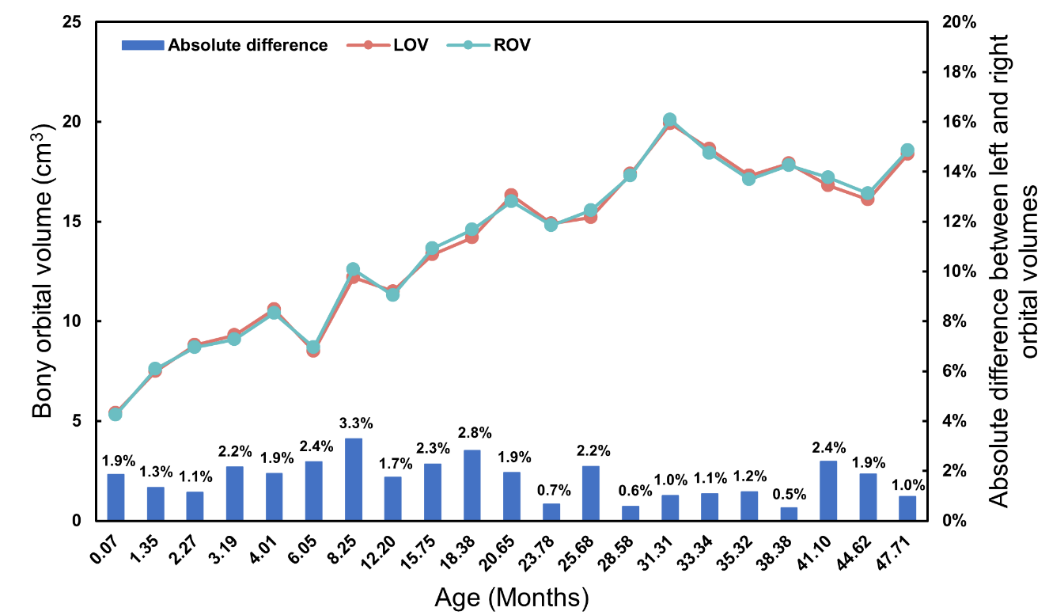
**

**Figure S6.** Sample distribution throughout the studied age range (0 to 48 months) reported in (A) 8 age groups with regular intervals. (B) 9 age groups with successive age intervals of varying duration.


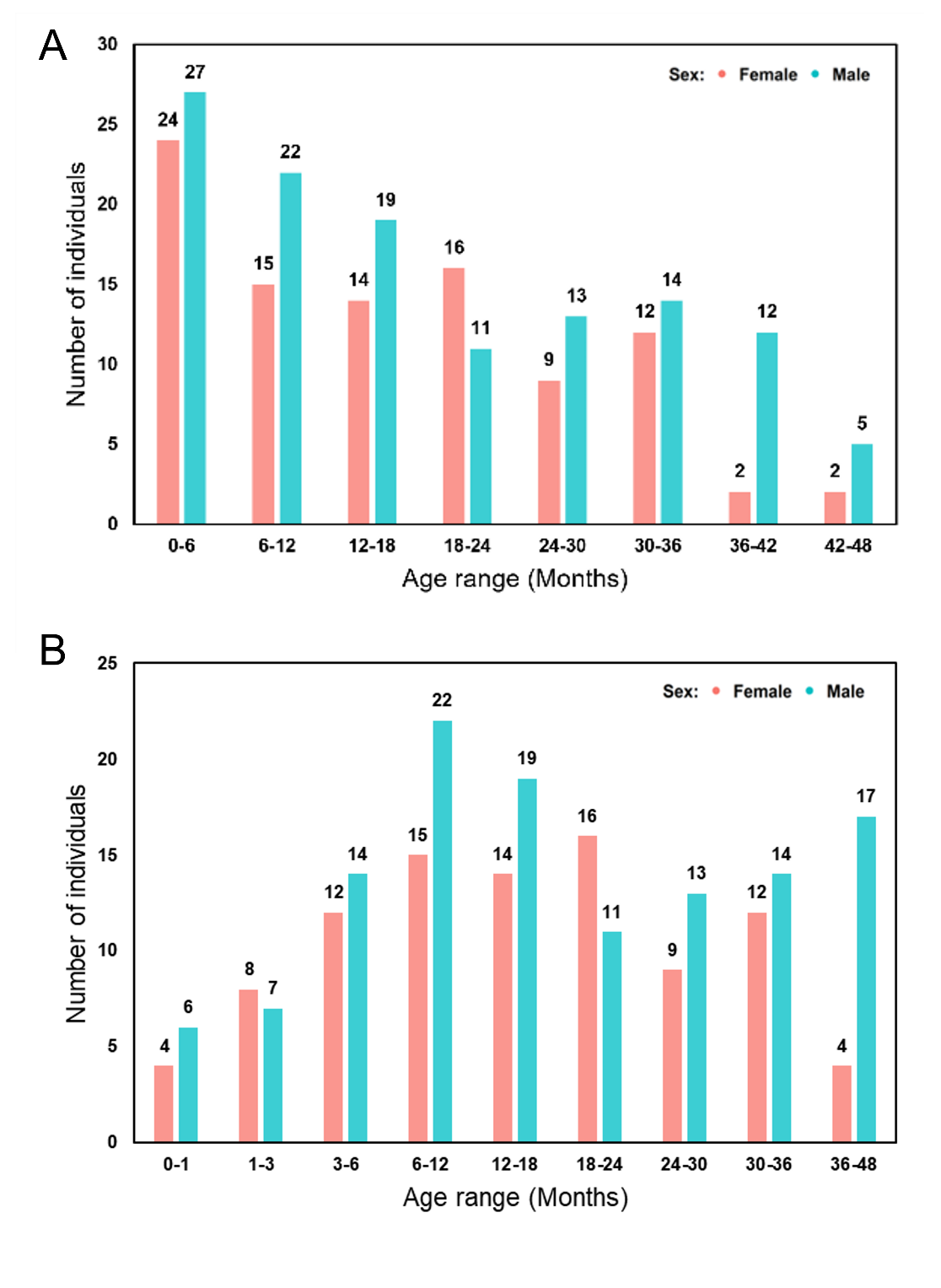


## References

S1. Howells, W. W. *Cranial variation in man: a study by multivariate analysis of patterns of difference among recent human populations*. vol. 67 (The Museum, 1973).

S2. Martin, R. & Knussmann, R. *Anthropologie: Handbuch der vergleichended Biologie des menschen*. vol. 1 (Gustav Fischer, 1988).

S3. Skrzat, J., Holiat, D. & Walocha, J. A morphometrical study of the human palatine sutures. *Folia Morphol (Warsz)* **62**, 123–127 (2003).

S4. Evteev, A., Anikin, A. & Satanin, L. Midfacial growth patterns in males from newborn to 5 years old based on computed tomography. *Am. J. Hum. Biol.* **30**, e23132 (2018).

S5. Wärmländer, S. K. T. S., Garvin, H., Guyomarc’h, P., Petaros, A. & Sholts, S. B. Landmark typology in applied morphometrics studies: What’s the point? *Anat Rec (Hoboken)* **302**, 1144–1153 (2019).

S6. Caple, J. & Stephan, C. N. A standardized nomenclature for craniofacial and facial anthropometry. *Int J Legal Med* **130**, 863–879 (2016).

S7. Libby, J. *et al.* Modelling human skull growth: a validated computational model. *J. R. Soc. Interface* **14**, 20170202 (2017).

S8. Lesciotto, K. M., Cabo, L. L. & Garvin, H. M. A morphometric analysis of prognathism and evaluation of the gnathic index in modern humans. *HOMO* **67**, 294–312 (2016).

S9. Gruber, P., Henneberg, M., Böni, T. & Rühli, F. J. Variability of human foramen magnum size. *Anat Rec* **292**, 1713–1719 (2009).

S10. Nikolova, S., Toneva, D. & Georgiev, I. A case of bipartite zygomatic bone. *Eur J Forensic Sci* **4**, 1 (2017).
